# Noradrenergic modulation of stress induced catecholamine release: Opposing influence of FG7142 and yohimbine

**DOI:** 10.1101/2024.05.09.593389

**Published:** 2024-05-09

**Authors:** Vladimir Visocky, Carleigh J. Turner, Matthew H Lowrie, Anthony Alibro, Fany Messanvi, Yogita Chudasama

**Affiliations:** Section on Behavioral Neuroscience, National Institute of Mental Health, National Institutes of Health, Bethesda, MD 20892, USA

## Abstract

In humans, loss aversion is sensitive to stress, and patients with neurological or psychiatric illnesses are particularly vulnerable to the detrimental effects of stress that lead to suboptimal life-altering choices. The basolateral amygdala (BLA) and nucleus accumbens (NAc) are stress sensitive brain areas that alter extracellular levels of norepinephrine (NE) and dopamine (DA), respectively. However, the dynamics of neurotransmitter release in these brain regions during stress has not been systematically explored. We used pharmacology and fiber photometric analysis to elucidate the impact of stress, DA and NE on brain activity during decision making behavior. Long-Evans rats were trained on an operant touchscreen decision-making task in which they chose between a *safe* stimulus that delivered a certain 50μl sucrose, or a *risky* stimulus that delivered either a ‘loss’ (10μl sucrose 75% of the time) or ‘win’ (170μl sucrose 25% of the time). Stress, induced by an inverse GABA_A_ agonist, FG7142, biased rats’ decisions towards safety due to increased loss sensitivity. The aversion to loss was blocked with co-treatment of the α2_A_ receptor antagonist, yohimbine. We also captured the rapid dynamic properties of stress induced changes in NE and DA release in the BLA and NAc, respectively. We discovered that these dynamics could be modulated with systemic injections of yohimbine by altering stress induced catecholamine release to optimize decision strategy and motivational state.

## INTRODUCTION

Everyday decisions involve uncertainty or situations in which the individual does not have sufficient knowledge to make an informed choice [1]. Such decisions carry ‘risk,’ and they involve facing trade-offs between options that promise safety and others that offer an uncertain potential for major gains and losses. In animals, as in humans, the canonical perspective is that decision-making is biased towards risk aversion, in a diverse range of animals including fish [2, 3], birds [4, 5] and bumblebees [6, 7]. It is thought this is due to the affective consequence of loss, or ‘loss aversion,’ an evolutionary conserved phenomenon known to be psychologically or emotionally more severe than an equivalent gain [8]. In humans, loss aversion is sensitive to stress [9-11], and patients with neurological or psychiatric illnesses are particularly vulnerable to the detrimental effects of stress thought to cause suboptimal or life-altering choices with long-term negative consequences [12-14]. Here we investigate the effect of stress neuromodulators on brain regions that modulate loss aversion in rats.

Many acute stressors increase extracellular concentrations of norepinephrine (NE) and dopamine (DA) in a number of brain regions indicating that stress can elicit widespread activation of catecholaminergic neurons [15]. Stress-susceptible brain regions that exhibit structural and/or functional alterations include the amygdala and nucleus accumbens [16-18], both of which contribute significantly to decisions that involve aligning reward gain with loss-sensitivity [19-23]. Stressful stimuli increase NE levels in the basolateral amygdala [24, 25] which produces inhibitory effects through α2 receptors to promote the stress response [16, 26, 27]. Thus, NE-modulation of stress in the amygdala could ostensibly influence loss aversion during decision making through stimulation of α2 receptors [19, 28]. Brain neurons containing and secreting dopamine (DA) are also activated during stress [29-31] causing a change in motivational state mediated by DA in the nucleus accumbens [32]. Consequently, stressed rats reduce their responding for food rewards [33, 34] and bias their decisions to low reward options due to an abnormal lack of motivation or anergia [33, 35]. Thus, both NE and DA can influence decisions under stress by affecting the decision strategy, motivation, and/or sensitivity to reward.

In the present study, we approach this topic with both pharmacology and fiber photometric analysis to elucidate the impact of stress, DA and NE on brain activity during decision making behavior. We induced stress pharmacologically with FG7142, a partial inverse GABA_A_ agonist known to induce anxiety-related behavioral and physiological responses in a variety of species, including humans [36]. We show that stress induced decisions that are biased towards safety can be blocked with the α2_A_ receptor antagonist, yohimbine. We also captured the rapid dynamic properties of stress induced changes in NE release in the basolateral amygdala (BLA) and compared it with DA release in the nucleus accumbens (NAc). We discovered that these dynamics could be modulated with the α2_A_ receptor antagonist to optimize motivated decision-making behavior.

## MATERIALS AND METHODS

### Subjects

Male Long-Evans rats (Envigo, Indianapolis, IN, USA) weighing 250-280g at the start of behavioral training were used for these studies. They were pair-housed in a temperature-controlled room (23.3 °C) under diurnal conditions (12:12 h light: dark). All testing occurred at a regular time during the light period. Rats were maintained at 90% of the free-feeding weight and water was available for at least 2hrs a day. All experimental procedures were approved by NIMH Institutional Animal Care and Use Committee, in accordance with the NIH guidelines for the use of animals.

### Decision-making behavior

**Figure 1.**
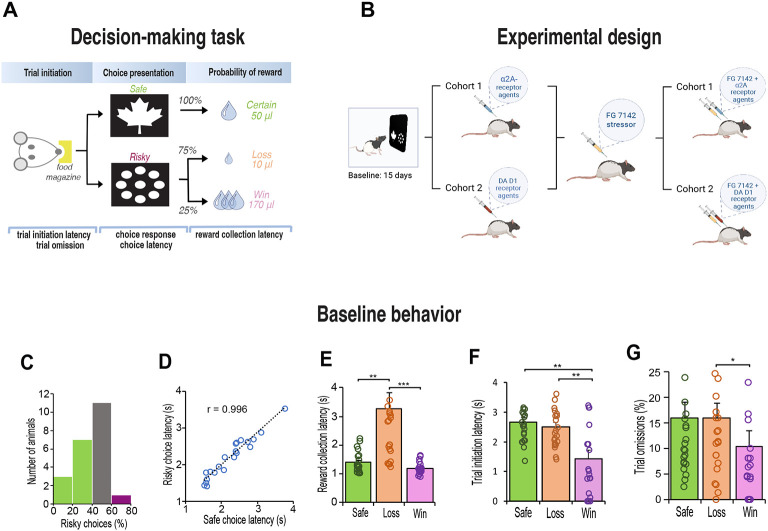
Schematic illustration of decision-making task, experimental design, and baseline behavior after 15 days of training. **A.** Rats initiated each trial with a nosepoke before choosing the ‘safe’ image (leaf) or the ‘risky’ image (circles). The safe image delivered a 50-μl reward. The risky image delivered a 10μl reward with 75% probability and a 170μl reward with 25% probability. The expected value of each choice was 50 μl of sucrose. **B**. After 15 days of training, rats (n = 22) were divided into two groups based on the treatment they received 30 minutes before the test. One cohort received a DA D1 agonist (SKF 81297) and antagonist (SCH 23390) and a second cohort was injected with an α2_A_ agonist (guanfacine) and antagonist (yohimbine). Drugs were injected in a counterbalanced manner. Following stable behavior, rats were injected with FG7142 to induce stress. Subsequently, each cohort was injected with the stressor combined with the dopaminergic or noradrenergic receptor specific drug. **C**. Histogram of risk preference. Green indicates risk-averse rats (> 40% risky choices); grey indicates indifferent rats (> 40% and < 60%); purple indicates risk-seeking rats (> 60%). **D.** Latency to choose the safe or risky option were equal. **E**. Reward collection latency was slower for reward loss reward (10-μl sucrose) for all rats (n = 22, Friedman test, χ^2^ = 42.91, p < 0.001; Safe vs Loss, Adj. p = 0.002; Win vs Loss, Adj p < 0.001). **F**. Latency to initiate a trial was always faster after a win (170μl sucrose) relative to after a safe/certain reward or reward loss (n = 22, one-way repeated ANOVA, interaction F_1,22_ = 16.913, p > 0.001; Safe vs Loss, p = 0.001; Win vs Loss, p = 0.002). **G**. After losing a reward, rats were more likely to omit next trial than after winning a high reward (n = 22, Friedman test, χ^2^ = 7.747, p = 0.021; Win vs Loss, p = 0.031). Data are mean and s.e.m. *** p < 0.001, ** p < 0.01, * p < 0.05.

Behavioral testing took place in eight automated touchscreen operant chambers (Lafayette Instrument Company, Lafayette, IN, USA) each comprising a standard operant chamber fitted with a touchscreen. Computer graphic stimuli composed of white geometric symbols on a black background were presented on a touch-sensitive monitor. Following habituation to the chamber, rats were trained to reliably initiate trials, touch the screen and collect 10% sucrose solution as a reward. During the test, rats chose between two different computer graphic stimuli presented on the left and right side of the touchscreen monitor (Fig. 1A). Each stimulus indicated differences in reward size and probability of outcome. Responses to the ‘safe’ stimulus (leaf) always resulted in the delivery of the small 50μl sucrose reward. Responses to the ‘risky’ stimulus (circles), delivered a small 10μl sucrose reward 75% of the time, or a large 170μl sucrose reward 25% of the time. The left/right positions of the risky and safe images were pseudorandomly determined thereby eliminating the potential confound of a side bias. Importantly, the expected value of the reward remained the same regardless of the animal’s choice. Each session started with 50 forced trials during which the safe or risky stimulus was presented to demonstrate the outcome associated with the stimulus. The remaining 200 trials were free choice trials in which rats could choose between both stimuli.

### Systemic pharmacology: Drug preparation and experimental design

All drugs were administered systemically i.p. and counterbalanced with vehicle. Doses of drugs were calculated as the salt and dissolved in the appropriate vehicle. Table 1 lists all drugs, dosages, and dissolving vehicle. Behavioral testing occurred 30 minutes after the injection. Drug test days were followed by a drug free day of no testing. Animals were then tested on the baseline schedule until performance stabilized before the next treatment. All drugs were purchased from Tocris (Tocris Cookson Inc., Ellisville, Missouri, USA). Dimethyl sulfoxide (DMSO) and 2-hydroxypropyl-β-cyclodextrin (HBC) as dissolving agents were purchased from Sigma-Aldrich (St. Louis, MO, USA).

Following stable baseline performance, we examined the animals’ choice of risk and safe options following changes to dopaminergic or adrenergic receptor activity (Fig. 1B). One cohort (n = 12) received injections of an adrenergic α_2A_-receptor agonist (guanfacine) and antagonist (yohimbine). A second cohort (n = 10) received injections of a dopamine D_1_ receptor agonist (SCH 23390) and antagonist (SKF 81297). Two weeks later, we induced stress in all animals by injecting them with a pharmacological stressor, FG 7142 (henceforth known as the stressor or FG stressor). This drug is a GABA_A_ inverse agonist which induces biochemical changes that mimic stress or anxiety strikingly similar to those elicited by mild aversive conditioning [37, 38], and therefore occurs independently of nociceptive stimuli. We then sought to block or reverse the effects of the stressor by co-injecting it with the dopaminergic or noradrenergic receptor specific drug.

### Fiber photometry: Viral injection and fiber implants

In a subset of pretrained animals (n = 9), we injected, unilaterally, a genetically encoded fluorescent NE sensor, GRAB_NE_ (pAAV-hSyn-GRAB_NE1m; Addgene #123308, gift from Yulong Li) into the basolateral amygdala (BLA), and a DA sensor, dLight (pAAV-syn-dLight1.3b; Addgene #135762, gift from Lin Tian) into the nucleus accumbens (NAc), to monitor NE and DA neural activity during stress induced decision-making. For all procedures involving local injections of injection and probe implantation, rats were anesthetized with isoflurane gas (5% induction, 2% maintenance) and secured in stereotaxic headholder (David Kopf Instruments, Tujanga, CA, USA). The scalp was retracted to expose the skull and craniotomies were made directly above the BLA (A/P −2.7 mm, M/L 4.9 mm, D/V −7.6) and NAc (A/P 1.7 mm, M/L 1.6 mm, D/V −7.3 mm). Viral injections were made using a pulled glass micropipette (WPI, USA) controlled by a Nanoliter 2020 injector (volume 300 nl at a rate of 100 nl/min). The virus was allowed to diffuse for 10 min before a slow withdrawal. Fiber optic cannulas (NA 0.66, 400-μm core diameter) were implanted 0.1 mm dorsal to the viral injection site (Doric Lenses, Canada). Cannulas were affixed with dental cement and stainless sterile screws to secure them in place.

### Fiber photometry recordings

Following a minimum of two-weeks after surgery, rats were re-trained to acquire stable decision-making performance (~ 4 weeks). We first monitored NE and DA activity in the BLA and NAc, respectively while animals engaged in the decision-making task. Subsequently, we examined NE and DA responses following systemic injections of vehicle, yohimbine (1mg/kg) and FG7142 (4mg/kg).

Fiber photometry data were acquired with the RZ10X processor integrated with software Synapse v.96 (Tucker-Davis Technologies, Inc. USA). The dLight and GRAB_NE_ (*F_465 nm_*) fluorescence signal was compared to signals for motion artefacts and photobleaching (*F_405 nm_*). Light was emitted from LEDs integrated with commercial Mini-cube fiber photometry apparatus (Doric Lenses) and a fiber patch-cord (400 μm core, 0.57 NA) connected to the implanted fiber-optic cannulas via a pigtailed rotary joint (Doric Lenses). The emitted signals were sent back to the mini cubes for filtration and detection by the integrated photosensors, and demodulated (210 Hz for 405 nm, and 330 Hz for 465 nm) in the Synapse software. In parallel, the RZ10X processor received time stamps of the behavioral events through a TTL breakout adapter (Lafayette Instruments, IN, USA). Raw fluorescence signals with behavioral time stamps were extracted into the Fiber photometry Modular Analysis Tool (pMAT) for further analysis [39]. A custom-made R code and pMAT were used to calculate delta F/F, Z-score and area under the curve (AUC) values used for analyses.

### Verification of fiber placement and viral expression

Rats were perfused transcardially, with a working solution of phosphate buffer saline (1X PBS) followed by 4% paraformaldehyde (PFA) dissolved in PBS. The brains were extracted and post-fixed in 4% PFA overnight at 4 °C, and then dehydrated in 30% sucrose in PBS for a week. The brains was then cryo-sectioned to 40 μm thickness using a freezing microtome (Leica Biosystems, USA). Sections were mounted on glass slides with Vectashield antifade mounting medium (Vector Laboratories, USA). High resolution images were taken with a microscope scanner (Axio Scan 7; Zeiss). Animals with misplaced cannulas or viral expression were excluded from analysis.

### Statistical analysis

The behavioral data were processed using custom-written programs in R and analyzed using SPSS Statistics 25.0 (IBM, Chicago, IL, USA). Incomplete data with sessions comprising less than 30% of free choices trials were not used for statistical analysis. All data were tested for normality and transformed accordingly before statistical significance testing. For comparisons between two groups *t*-tests were used. In cases when the data did not fit the assumptions of the test, the nonparametric Mann–Whitney or Wilcoxon matched-pairs tests were used. For repeated measures ANOVA, data was assessed for homogeneity of variance using Mauchly’s sphericity test. When this requirement was violated for a repeated measures design, the F term was tested against degrees of freedom corrected by Greenhouse–Geisser to provide a more conservative p value for each F ratio. Otherwise, nonparametric Friedman’s test (χ^2^) was applied with differences compared with posthoc Wilcoxon signed-rank tests (Z) adjusted with a Bonferroni correction. Pearson correlation (r) was used to describe the linear relationship between two correlated variables.

## RESULTS

### High sensitivity to loss impacts motivational state

After 3 weeks of testing, rats displayed consistent choice preferences across three consecutive days (χ^2^_(2)_ = 0.302, p = 0.86). Like human choice behavior [40], the rats were either indifferent in their choices (n=11) or exhibited stable risk-aversion (n=10). Only one rat exhibited risk taking behavior by choosing the risky option more than 60% of the time (Fig. 1C). Regardless of their choice, the speed at which the animals made their response did not differ between the safe or risk option (t_(21)_ = 0.97, p = 0.34), and correlated strongly (Pearson r_(21)_ = .97, p < 0.001) such that the latency to choose the safe or risky option were equal (Fig 1D). However, their motivation to collect reward was highly influenced by their choice, especially following selection of a risky option that led to a loss (χ^2^_(2)_ = 42.09, p < 0.001). Posthoc tests confirmed that the latency to collect reward was twice as fast following delivery of the safe-certain reward (Z = 3.47, p = 0.002) and a risk-win reward (Z = 6.48, p < 0.001) relative to a risk-loss (Fig. 1E). Their motivation to initiate the next trial was also influenced by the outcome of the choice; after choosing the risky option that led to a reward loss, the speed to initiate the next trial was substantially slower than following a win (F _(1, 23)_ = 16.91, p < 0.001, Fig. 1F), and in many cases resulted in trial omissions (Z = 2.56, p = 0.031, Fig. 1G).

### α_2A_ – adrenoceptors modulate sensitivity associated with reward loss

**Figure 2.**
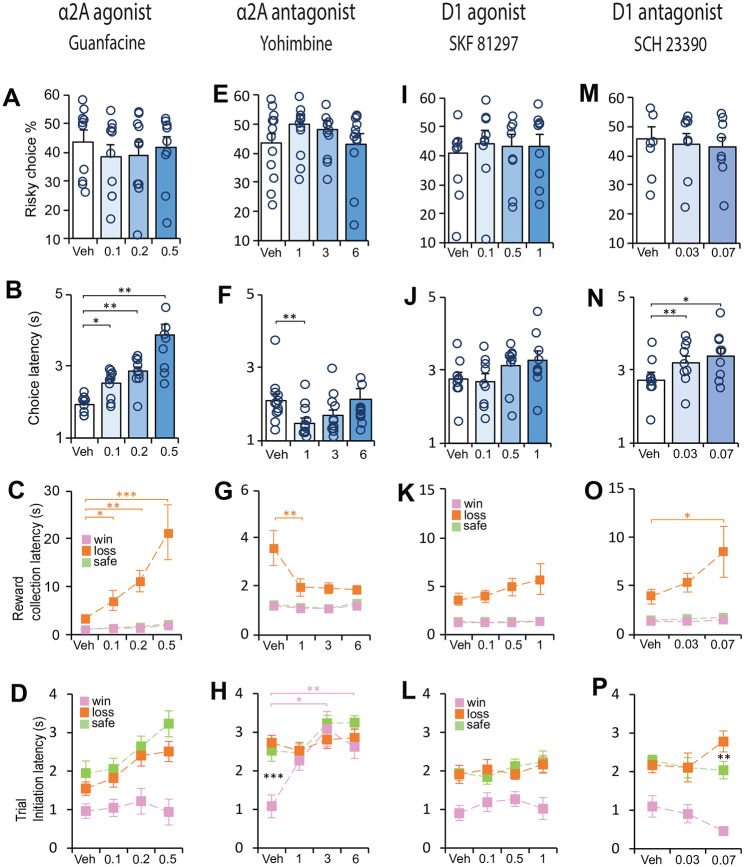
The effect of DA D1 and α2_A_ agonist and antagonists on decision making behavior. Graphs **A, E, I, M** show that risky choices were not affected by DA D1 and α2_A_ drug treatments. Graphs **B, F, J, N** show that guanfacine and SCH 23390 treatment slowed down decision speed (Guanfacine, n =10, one-way repeated ANOVA, interaction F_3,27_ = 24.512, p = 0.004; Veh vs Low, p = 0.012; Veh vs Med, p = 0.002; Veh vs High, p = 0.002; SCH 23390, one-way repeated ANOVA, interaction F_1,10_ = 11,05, p = 0.006; Veh vs Low, p = 0.002; Veh vs Med, p = 0.024). In contrast, treatment with yohimbine made rats faster in their response speed (n = 12, Friedman test, χ^2^ = 15.5, p = 0.001; Veh vs Low, p = 0.01). **C, G, K, O** show that reward collection latencies were significantly increased with guanfacine and SCH 23390 (Guanfacine, n =10, two-way repeated ANOVA, interaction F_3,23_ = 14,41, p < 0.001; Veh vs Low, p = 0.017; Veh vs Med, p = 0.006; Veh vs High, p < 0.001; SCH 23390, n = 9, two-way repeated ANOVA, interaction F_2,13_ = 14,41, p = 0.021; Veh vs Med, p = 0.031). Opposite to guanfacine, yohimbine made rats faster after loss (yohimbine, n = 10, two-way ANOVA, interaction F_2,47_ = 9.85, p = 0.002; Veh vs Low, p = 0.005). **D, H, L, P** Trial initiation latency was generally faster after winning a high reward except with yohimbine (Yohimbine, n = 12, two-way repeated ANOVA, interaction F_3,33_ = 4.84, p < 0.001; Vehicle, one-way repeated ANOVA, interaction F_2,22_ = 24.4, p < 0.001; Win vs Loss, p < 0.001 ; Yohimbine 1 mg/kg, one-way repeated ANOVA, interaction F_2,22_ = 0.457, p = 0.639. Low – low concentration of drug. Med – medium concentration of drug. High – high concentration of drug. All concentrations are in mg/kg. Data are mean and s.e.m. *** p < 0.001, ** p < 0.01, * p < 0.05.

We next examined how choice for safe or risky options were modulated by noradrenergic drugs that acted on the α_2A_-receptor. There is some evidence that stimulation of α_2A_-receptors affects some forms of decision making even in normal animals [41, 42]. To test this possibility, we first injected a cohort of trained rats with low, medium, and high doses (Table 1) of guanfacine (α_2A_-receptor agonist) and yohimbine (α_2A_-receptor antagonist), in separate sessions, each counterbalanced with vehicle. Neither drug had any impact on the animals’ preference for the choice at any dose (guanfacine: χ^2^_(3)_ = 4.09, p = 0.252; yohimbine: χ^2^_(3)_ = 5.5, p = 0.139; Fig. 2A,E), but substantially altered the animals’ sensitivity to loss and motivational state, in opposite directions. In general, guanfacine demotivated the animals by slowing them down without impacting their motoric abilities. For example, their latencies to make a choice increased with higher doses (F_(3, 27)_ = 24.512, p = 0.001; Fig. 2B), but their latencies to collect reward depended on the reward outcome (F_(3, 23)_ = 14.41, p < 0.001). When the animal chose the risky option and experienced a reward loss, these animals were disproportionally slower in collecting the reward which got worse with increasing dose (F_(3, 27)_ = 23.811, p < 0.001; Fig. 2C). The same animals, however, were distinctly fast to collect reward following a choice response that led to a win or a safe-certain reward at all doses. Thus, the long latencies could not be explained by mere sedation, but a specific sensitivity to reward loss. Similarly, the latency to initiate a trial following the different reward outcomes was impacted across all doses (F_(3, 25)_ = 2.93, p = 0.056; Fig. 2D).

Opposite to the effects of guanfacine, antagonizing the **α_2A_**-receptors with yohimbine made the animals almost insensitive to the reward outcomes; their choice strategy remained constant (Fig. 2E), they were faster to make a choice response especially at the low dose (χ^2^_(3)_ = 15.5, p = 0.001; Fig. 2F) and comparatively faster than guanfacine (compare with Fig. 2B), and failed to discriminate between reward loss and reward win since they were equally fast to collect rewards with both outcomes (F_(2,18)_ = 7.75, p = 0.005; Fig. 2G). Moreover, Fig. 2H shows that with yohimbine, the animals were so insensitive to the different reward values that their motivation to initiate the next trial was identical for all reward outcomes including after reward losses (F_(2, 22)_ = 0.46, p = 0.64; Fig. 2H), but not with vehicle (F_(2, 22)_ = 24.4, p < 0.001).

### Blocking dopamine D_1_ receptors reduces motivational state

Since many psychiatric disorders characterized by risky decision making are associated with dysregulated dopamine transmission [43, 44], in a separate cohort of rats, we also examined the effects SKF 8129 (dopamine D_1_ receptor agonist) and SCH 23390 (dopamine D_1_ receptor antagonist) for comparison. We found that the D_1_ agonist had no major impact on decision making behavior, and that aspects of motivation including speed of response and reward collection following safe or risky choices were within the normal range (Fig. 2I-L). In contrast, the D_1_ antagonist, while not affecting the animal’s choice behavior (Fig. 2M), did alter their motivation, which in some way was similar to the effects of the α_2A_-receptor agonist, guanfacine. First, these animals were slow in their response for both doses relative to vehicle (0.03 mg/kg, p = 0.002; .07 mg/kg, p = 0.024; Fig. 2N). Second, there was a dose dependent increase in reward collection latency but only following a choice that led to a reward loss (F_(2,13)_ = 5.40, p = 0.021; Fig. 2O). As expected, motivation for initiating a trial in this cohort of animals was relatively fast following a win (Fig. 2P), especially at the highest dose (0.07 mg/kg; F_(2,16)_ = 50.312, p > 0.001).

### Yohimbine blocks stress-induced sensitivity to reward loss

**Figure 3.**
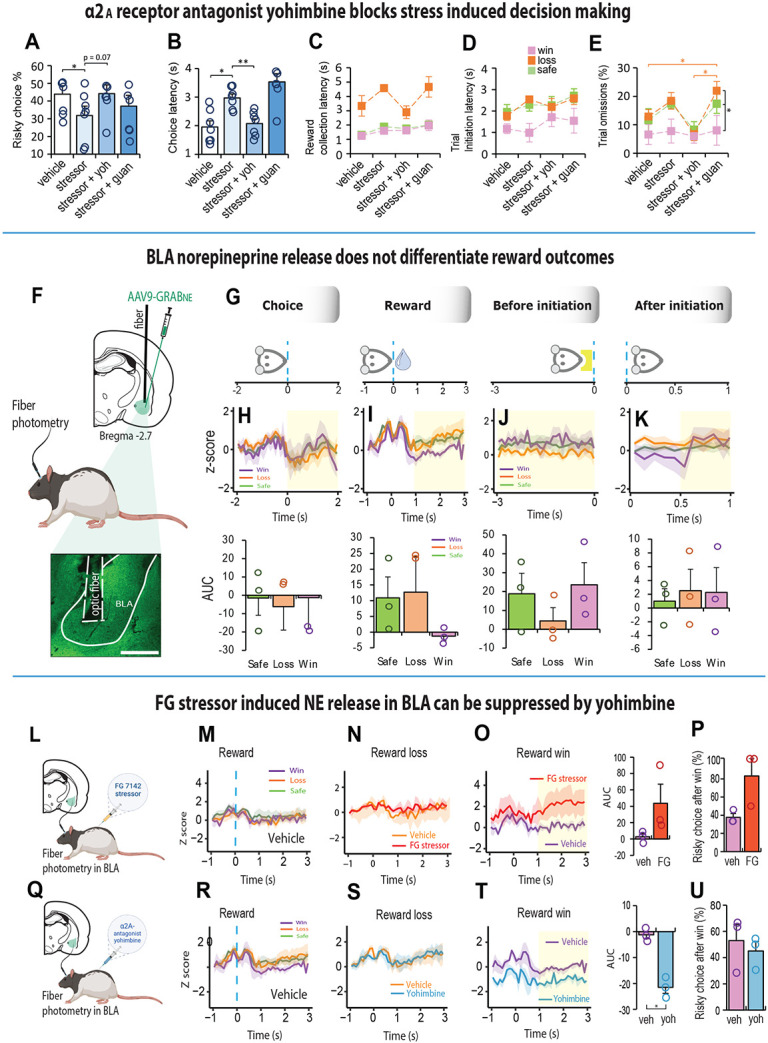
Stress induced NE release in BLA is modulated by activating α2_A_ – adrenoceptors. **A-E.** Pharmacological stress induced by an i.p. injection of 4 mg/kg of FG 7142 reduced risky choices and increased their choice latency, while co-treatment of FG 7142 with yohimbine reversed these effects; rats were faster in their choices and increased their choice of risky options to a level of indifference (Risky choice%, n = 7, one-way repeated ANOVA, F_3,18_ = 6.408, p = 0.004; Veh vs FG7142, p = 0.016; FG7142 vs FG7142 + Yoh, p = 0.07. Choice latency, one-way repeated ANOVA, F_3,18_ = 25.859, p < 0.001; Vehicle vs FG7142, p = 0.026; FG7142 vs FG7142 + Yoh, p = 0.008). Combined treatment of FG 7142 with guanfacine made rats slow and omit more trials after a reward loss (Choice latency, one-way repeated ANOVA, F_3,18_ = 25.859, p < 0.001; Veh vs FG7142 + Guan, p < 0.001; FG7142 vs FG7142 + Guan, p = 0.003. Trial omissions, two-way repeated ANOVA, F_6,36_ = 2.827, p = 0.023; Veh vs FG7142 + Guan, p = 0.049; FG7142 + Guan vs FG7142 + Yoh, p = 0.037). **F**. Schematic representation of AVV9-GRAB_NE1m expression in the BLA and histology portraying optic fiber placement above the injection site. **G**. Schematic illustration showing how NE signal was aligned to four specific events in the trial. During the choice phase, the rat touched the screen and moved towards reward collection. During the reward collection phase, rat entered the food magazine to collect the reward. Before initiation phase describes NE activity 3 second before the next trial was initiated. After initiation phase shows 1 second of NE activity after trial initiation, during which rat moves towards touchscreen for the next choice. **H, I, J, K** Top row. Mean noradrenaline signal during choice, collection, after initiation and before initiation phases (n = 3). The dynamics of NE release are different for reward wins (purple), safe/certain rewards (green) and reward loss (orange). The yellow shaded area assigns the period used for the area under the curve analysis (AUC). Bottom row. Signal quantification for each phase. **L**. Schematic illustration of fiber photometry probe in BLA injected with FG7142 administration. **M.** Mean NE signal in the BLA during reward collection following vehicle injection (n = 3). **N**. NE signal in BLA following vehicle (orange) and FG7142 (red) treatment (n = 3) are indistinguishable. **O.** Mean NE signal after win in FG7142 (red) increased relative to vehicle (purple); t2 = 2.02, p = 0.181). **P**. The increase in NE signal after collecting a high reward was associated with a higher propensity to make risky choices (t2 = 2.675, p = 0.11). **Q**. Schematic illustration of fiber photometry probe in BLA injected with yohimbine. **R.** Mean NE signal in the BLA during reward collection following vehicle injection (n = 3). NE signal did not differentiate between win (purple), safe (green) and loss (orange) reward outcomes. **S**. NE signal for reward collection after a reward loss was indistinguishable between vehicle (orange) and yohimbine (blue). **T.** NE signal in BLA after reward win in yohimbine treated rats reduced relative to vehicle (purple); (t2 = 9.407, p = 0.011). **U.** The NE signal reduction did not influence the subsequent choice . Data are mean and s.e.m. AUC – area under the curve. Veh – vehicle. FG – FG7142. Yoh – yohimbine. Scale bar 500 μm. ** p < 0.01, * p < 0.05.

We next examined if decision-making was sensitive to physiological stress. Due to the habituation caused by repeated exposure to acute stress, we gave rats a systemic 4mg/kg dose of a pharmacological stressor known as FG 7142 (henceforth known as stressor or FG stressor). This drug is known to mimic the effects of uncontrollable stress linked to anxiety [45] and increases catecholamine turnover in various limbic associated areas including the BLA and NAc [46-48] thought to influence choice behavior in humans and animals [49]. The stressor veered rats’ choices towards safety (F_(3, 18)_ = 6.408, p = 0.004; veh vs stressor, p = 0.016; Fig. 3A) and made them slower in their response (F_(3, 18)_ = 25.859, p < 0.001; veh vs stressor, p = 0.026; Fig. 3B) suggesting they were less motivated to engage in risk taking behavior. Apart from an increased sensitivity to reward loss, other aspects of motivation were relatively intact (Figs. 3C-E). In humans, however, the co-administration of the stress hormone hydrocortisone with yohimbine diminishes loss aversion [11]. To test this possibility, we co-injected the FG stressor with yohimbine and found, that their concurrent actions tended toward reduced loss aversion in rats relative to the stressor alone (F_3, 18_) = 6.408, p = 0.004; FG vs FG + Yoh, p = 0.07; Fig. 3A), and made them noticeably faster in their choices (p = 0.008; Fig. 3B). It also increased their motivation to collect low rewards, initiate trials and reduced the number of omissions after a reward loss (Figs 3C-E). In contrast, the behavior associated with combined guanfacine and the FG stressor was equivalent to the stressor alone (all p>0.05, NS).

### Stress induced NE release in BLA can be suppressed by blocking α2_A_ - adrenoceptors

We next asked if the fluorogenic NE reporter GRAB_NE_, could capture the rapid dynamic properties of NE release for decisions that led to different reward outcomes. We focused on the basolateral amygdala (BLA) because it is diffusely innervated by NE projecting neurons from the locus coeruleus [50-52], and NE levels in the BLA increase with presentation of stressful stimuli [24, 25]. We injected rats with an AVV that expressed GRAB_NE_ in the BLA and implanted an optic fiber above the injection site (Figs. 3F, S1A). After a minimum of 4 weeks to allow the expression of the viral sensor, rats were placed in the test chambers and assessed on their decision-making while recording the GRAB_NE_ fluorescence. The fluorescent signal was aligned to four specific events in the trial: the choice, reward collection, before trial initiation and after trial initiation (Fig. 3G). Changes in NE release were not observed in the BLA during the decision itself such that the kinetics of the NE response were relatively equivalent before and after the choice (Fig. 3H). A double activation of signal for NE release in the BLA was observed just before and immediately after reward collection regardless of the trial outcome (Figs. 3I, S2A-C). Subsequently the signal declined especially for choices that led to large reward wins. The signal for the remaining trial events did not differentiate the reward outcomes. Thus, NE release in the BLA does not correlate directly with choices influenced by risk or uncertainty. However, since yohimbine and the FG stressor altered the animal’s sensitivity to reward outcome when injected systemically (Figs. 3A-E), we measured NE signal in the BLA following the injection of both these agents (Figs. 3L, Q).

We found these drugs to have opposing effects on NE release but only when we aligned the signal to reward collection (Figs. 3L-U). While the stressor had no impact on the NA response for reward losses (Fig. 3N), it increased the NE signal following a large reward win (Fig. 3O; t_2_ = 2.02, p = 0.181), and this was associated with a higher propensity to make risky choices after a win (t_2_ = 2.675, p = 0.11; Fig. 3P). Conversely, systemic yohimbine reduced NE release in the BLA relative to vehicle when the choice led to a large reward win (t_2_ = 9.407, p = 0.011) but this did not influence the subsequent choice (Fig. 3Q-U). The BLA-NE signal did not change for either drug when the reward collection followed a loss (Fig. 3N,S). These results suggest that blocking the activation of α2A – adrenoceptors can potentially suppress or shift the effects of stress induced risky behavior by making the animal choose safer options.

### Stress induced DA response in the NAc is modulated by α2_A_ – adrenoceptors

**Figure 4.**
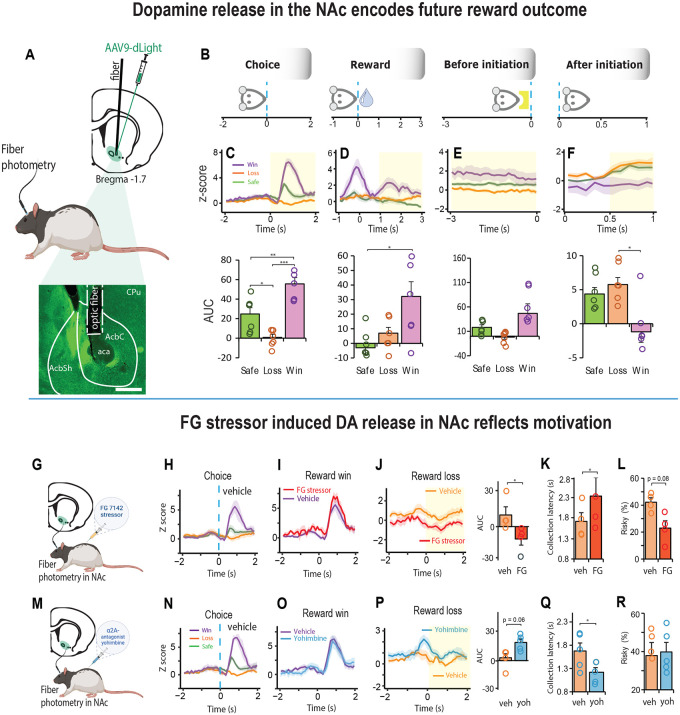
Stress induced DA release in the NAc reflects motivation during decision-making. **A** Schematic representation of an AVV9-dLight expression in the NAc and histology portraying optic fiber placement above the injection site. **B**. Schematic illustration showing how DA signal was aligned to four specific events in the trial: During the choice phase, the rat touched the screen and moved towards reward collection. During the reward collection phase, rat entered the food magazine to collect the reward. Before initiation phase describes NE activity 3 second before the next trial was initiated. After initiation phase shows 1 second of NE activity after trial initiation, during which rat moves towards touchscreen for the next choice. **C - F.** Mean DA signal in NAc during choice response, reward collection, after trial initiation and before trial initiation phases (n = 5). **C.** Strength of DA release varied systemically as a function of reward value such that DA release was high when there was an increase in future reward value (a reward win: purple), inhibited when there was decrease in future reward value (a reward loss: orange), and intermediate when the future reward was low but certain (green); (one-way repeated ANOVA, F_2, 10_ = 57.39, p < 0.001; Win vs Loss, p < 0.001; Win vs Safe, p = 0.002; Loss vs Safe, p = 0.017). Shaded yellow area assigns the period for the area under the curve analysis (AUC). **D.** After win reward collection, dopamine dynamics are greater than after safe/loss reward collection (one-way repeated ANOVA. F1, 5) = 12.18, p = 0.014; Win vs Loss, p = 0.097; Win vs Safe, p = 0.029). **F.** Interestingly, next trial animal start with low dopamine signal if the previous choice was win (one-way repeated F_2,10_ = 8.05, p = 0.008; Win vs Loss, p = 0.024; Win vs Safe, p = 0.054). **G.** Schematic representation of dopamine signal collection in NAc after FG7142 administration. **H.** Mean dopamine signal during choice phase after vehicle injection (n = 4). **I.** Mean DA signal after win were similar between vehicle (purple) and FG7142 (red) **J.** Mean DA signal after loss in FG7142 pretreated rats was strongly reduced relatively to vehicle (t_3_ = 4.299, p = 0.023). **K-L.** FG 7142 caused DA signal reduction was associated with reduced loss collection latency (t_3_ = 3.875, p = 0.03) and increase in choices towards safety (t3 = 2.6, p = 0.08). **M.** Schematic representation of dopamine signal collection in NAc after yohimbine administration. **N.** Mean dopamine signal during choice phase after vehicle (HBC, see methods) injection (n = 5). **O.** Mean DA signals after loss during vehicle and yohimbine treatment are indistinguishable **P.** Mean DA signal after loss in yohimbine pretreated rats was higher relatively to vehicle (yellow). Signal quantification between vehicle and yohimbine (AUC, t_4_ = 2.55, p = 0.063). **Q-R.** Decrease in dopamine signal during loss was associated with reduced collection latency but did not affect rat’s strategy (loss collection latency, t_4_ = 2.783, p = 0.049). Data are mean and s.e.m. AUC – area under the curve. Veh – vehicle. FG – FG7142. Yoh – yohimbine. Scale bar 500 μm. *** p < 0.001, ** p < 0.01, * p < 0.05.

We also asked if stress induced changes in motivation could be differentially modulated by dopamine since the D_1_ antagonist reduced motivational state. We first co-injected the FG stressor with a dopamine D_1_ agonist (SKF 81297) or antagonist (SCH 23390). Unfortunately, most of the animals were unable to tolerate the drug combination. We lowered the dose of the stressor to 1mg/kg to increase the sample size but found that the low dose was insufficient to alter the animals’ normal range of behavior (Fig. S3). There is much evidence however, that stress has profound effects on the mesoaccumbens dopamine system [30, 31, 53] and that projections from dopaminergic nuclei to the nucleus accumbens (NAc) play an important role in motivated decision-making behavior [54, 55]. Accordingly, we first recorded changes in fluorescence of the genetically encoded dopamine (DA) sensor, dLight expressed in the NAc to examine changes in the DA response during decision-making (Fig. 4A, S1B). The highest peak of DA release in the NAc was observed after the choice was made, and it related to the value of the future reward (Figs. 4B-D, S2D-F). Thus, DA release was high when there was an increase in future reward value (a reward win), inhibited when there was decrease in future reward value (a reward loss), and intermediate when the future reward was low but certain (Choice: F _(2, 10)_ = 57.39, p < 0.001). Moreover, the DA response for the winning choice peaked high during reward collection and elevated again *after* reward collection (F _(1, 5)_ = 6.95, p = 0.031; Fig. 4D). High dopamine release after a win persisted to some degree until the animal initiated the next trial (before initiation: F _(1, 5)_ = 3.3, p = 0.079; Fig. 4E), which could potentially explain the high motivational state characterized by faster trial initiations and reduced omissions (Fig 1E-G). After the next trial was initiated, the DA signal reversed; the previous reward wining outcome resulted in a reduction in DA release whilst the previous low reward outcomes enhanced DA release in the NAc (after initiation: F _(2, 10)_ = 8.06, p = 0.008; Fig. 4F).

We next examined changes in DA release in the NAc following systemic injections of the FG stressor (Fig. 4G-L) and found that it had no impact on the DA signal for choices that led to a reward win (Fig. 4 I). However, for those choices that led to a reward loss, the DA signal declined relative to vehicle (Fig. 4J). Moreover, there was an associated increase in loss sensitivity characterized by slower collection of low rewards and increased aversion to risk (AUC, t_3_ = 4.299, p = 0.023; loss collection latency, t_3_ = 3.875, p = 0.03; % risky choice, t_3_ = 2.6, p = 0.08; Fig. 4K, L). We then discovered that these effects could potentially be reversed by blocking α2A - adrenoceptors with yohimbine (Fig. 4M-R). This not only increased DA release in response to reward loss (Fig. 4P), it increased the rat’s motivation by making them faster to collect a low reward, without affecting their decision strategy (Fig. 4Q,R; AUC, t_4_ = 2.55, p = 0.063; loss collection latency, t_4_ = 2.783, p = 0.049).

## DISCUSSION

There were three main findings: 1) stress induced decisions that were biased towards safety were blocked with the α2_A_ – adrenoceptor antagonist, yohimbine, 2) yohimbine can modulate stress induced NE release in the BLA to shift risk preference, and 3) yohimbine can modulate stress induced DA in the NAc to alter strategy and motivational state. Together, these findings demonstrate that stress induced changes in catecholamine release can be modulated by activation of α2_A_ – adrenoceptors to influence loss sensitivity, choices and motivation.

### Opposing influence of α2_A_ adrenoceptor activation

One major finding of our study is that NE modulation at α2_A_ adrenoceptors has a powerful influence on how reward loss is incorporated into the animals’ decision. We found that loss aversion was disproportionately high in animals injected with the α2_A_ agonist, guanfacine; they were slow in making choices, collecting their rewards, and initiating trials, but only when their choice resulted in a low reward outcome. When the choice outcome resulted in a high reward, rats showed normal levels of motivation and speed of response even at the high dose confirming that the demotivating effects of systemic guanfacine following reward loss were not due to sedative effects of the drug. Guanfacine, through its postsynaptic actions in the prefrontal cortex, is known to enhance cognitive functions that subserve attention and working memory [56, 57]. One possibility is that while guanfacine made the α2_A_ adrenoceptor highly sensitive to decisions that lead to major losses, it functioned to enhance or focus attention most efficiently towards choice outcomes with positive consequences. That DA D_1_ receptor antagonism with SCH 23390 produced behavioral effects similar to that of guanfacine is suggestive of the idea that risk taking for potential losses involves both noradrenergic and dopaminergic receptor mechanisms.

In contrast, when the α2_A_ receptors were antagonized with yohimbine, these same animals became hyper-motivated showing no evidence of loss aversion; they were so eager and fast in their latencies, they failed to discriminate the different reward values associated with the choice outcome. Consequently, these animals worked equally fast for all trials even when their chosen option lead to a loss. While it is possible that the fast, indiscriminate responding with yohimbine was caused by an impulsive-like state [58-60], it did not make them risk prone. In fact, despite the robust alterations in the animals’ motivational state, neither yohimbine nor guanfacine, when systemically administered alone, had any impact on the animal’s decision strategy.

### The effect of pharmacological stress on decision-making

Several lines of evidence support the idea that acute and chronic stress increases reward sensitivity and bias choices towards safer options [61-63]. We discovered that the systemic administration of two well-known pharmacological stressors, FG 7142 and yohimbine, have opposing effects on decision-making behavior. Yohimbine mimics stress by stimulating inhibitory α2_A_ adrenergic presynaptic autoreceptors thereby increasing noradrenergic transmission [64-66]. In contrast, the stressor FG 7142 decreases GABAergic transmission through allosteric inhibition at the GABA_A_/benzodiazepine receptor complex [36, 67]. Unlike yohimbine, the FG stressor increased rats’ sensitivity to reward loss, such that animals avoided incurring losses by mostly choosing the option that delivered the safe/certain reward. Intriguingly, when combined, yohimbine reversed the detrimental effect of the FG stressor on loss sensitivity and shifted their decision strategy. Similar results are observed in humans when hydrocortisone is combined with yohimbine [11, 68]. The ability of yohimbine to block the effects of FG 7142 reveal a critical interaction between the GABA_A_/benzodiazepine receptor complex and the noradrenergic system. FG 7142, by antagonizing GABA_A_ receptors, reduces the inhibitory effect of GABA on neuronal activity thereby increasing their excitability. Reduced inhibitory synaptic neurotransmission alone can induce anxiety-like behavior [69]. In addition, FG 7142 acting on GABA_A_ receptors increase NE turnover in the several brain regions including the amygdala, nucleus accumbens and the prefrontal cortex [70, 71], as the locus coeruleus, which houses the largest group of NE neurons, is under inhibitory control of the GABA_A_ receptor [72]. Thus, the combination of both, FG 7142 and yohimbine, by reducing GABAergic inhibition and increasing noradrenergic activity would be expected to enhance stress mediated anxiety-like responses which could potentially impair decision strategy. Surprisingly, we found this not to be the case. Instead, the combination of the two stressors shifted the decision strategy to control levels while improving decision-making speed and motivation. In other words, the joint effect of both stressors was beneficial, not detrimental, suggesting that yohimbine was able to block the effects of FG 7142-induced hyperactivity of BLA-NE neurons [73]. It also suggests that activating the α2_A_ adrenoreceptor may well facilitate the positive influence of GABA mediated benozdiazepines on stress related anxiety.

### α2_A_ adrenoceptor modulation of stress induced catecholamine release

Although systemic injections of the FG stressor shifted rats’ preferences for low-risk choices, its impact on NE activity in the BLA was markedly different. The photometry trace revealed a high NE signal in BLA neurons for choices that led to reward wins, which further inclined the rat to make high-risk choices. The high NE signal is consistent with the general finding that stressful stimuli increase NE release in the BLA [24, 48, 74], In fact, when rats were not stressed, there was very little variation in NE release in the BLA suggesting that basal levels of BLA-NE have little impact on decision-making under risk. Notably, the NE signal did not change when choices resulted in a reward loss suggesting that NE in the BLA, while sensitive to emotionally stressful or fearful contexts [75, 76], may not encode the affective consequence of loss, or ‘loss aversion.’ This difference may be due to the nature of the stressor or its intensity which differentially impacts the activity of noradrenergic neurons in different brain regions [77-79]. Since benzodiazepine receptor binding is altered by stress [80, 81], and NE acts on GABAergic cell populations indigenous to the BLA region [82, 83], the inclination to be risky may be related to impaired NE modulation of GABA transmission in the BLA. Our finding that systemic injections of yohimbine reduced the BLA-NE signal without affecting decision strategy is consistent with this hypothesis.

We note that unlike other published reports [84, 85], the effects of the DA D_1_ agonist/antagonist on decision making were modest at best, but not uncommon [86-88]. In our case, the DA D_1_ agonist and antagonist had a noticeable effect on motivation which was further supported with the photometric analysis of DA release in the NAc. In the absence of stress, the DA signal in the NAc encoded both, the reward value associated with the choice, as well as the magnitude of the high reward which was sustained for some time after reward collection. Notably, the size of the NAc-DA signal positively correlated with the rat’s motivation (Fig. S4), a finding contrary to that of Eshel et al., [54] where motivation, characterized by the willingness to overcome the cost of working in mice, was found to be negatively correlated with NAc-DA activity. In the present study, the sensitivity to reward loss exacerbated when the animal was stressed with FG 7142. This resulted in a reduced DA signal in the NAc, which disappeared when treated with yohimbine. In fact, with yohimbine, the NAc-DA signal was high even *before* the choice was made, and this predicted the animals increased motivation to decide faster on their options and collect their rewards, even for low rewards. Thus, although DA activity in the NAc is sensitive to experienced loss during stress, we found that the reduced motivation exhibited by these animals for low reward choices can be potentially countered with an α2_A_ adrenoreceptor interaction with GABA_A_ receptors in the NAc.

### Concluding remarks

We applied methods of behavior, psychopharmacology and neurophotometrics to measure the neural dynamics of stress modulators in brain regions that affect decision-making in rats. Like humans, we showed that rats imitate the loss aversion effect and its associated outcome-based motivations. Our results support the proposal that stress induced changes in catecholamine release in the BLA and NAc can directly influence loss sensitivity, choices and motivation, which can be modulated by the α2_A_ adrenoreceptor, yohimbine. Stress associated catecholamine release exacerbates vulnerability to a variety of clinical conditions in which patients engage in decision making involving risks and rewards. Elucidating the complex interplay between neuromodulatory circuits that mediate this form of decision making will improve our understanding of the dysfunctional pathways linking the stress response to suboptimal life-altering choices.

## Supplementary Material

Supplement 1 **Figure S1. Photometry fiber placement in nucleus basolateral amygdala and nucleus accumbens. A.** Green dots in coronal sections represent tips of the fibers used to record noradrenaline dynamics in amygdala. **B.** Green in coronal sections represent tips of the fibers used to record dopamine dynamics in nucleus accumbens.
**Figure S2. Dopamine and noradrenaline dynamics during choice and collection in single animal. A.** Schematic representation of collection phase. During collection phase, rat entered the magazine to collect the reward. **B.** Peri-event time histogram visualize noradrenaline activity in each trial during safe, loss and win collection. **C.** Averaged change of fluorescence intensity during safe, loss and win collection. **D.** Schematic representation of choice phase. During choice phase rat approached the screen, touched the screen, and moved towards reward collection. **E.** Peri-event time histogram visualize dopamine activity in each trial during safe, loss and win choice. **F.** Averaged change of fluorescence intensity during safe, loss and win choice.
**Figure S3. Involvement of D1 receptor in stressed decision making. A- E.** FG 7142 co-injection with D1 agents did not introduce any significant changes in the rat’s decision-making behavior. Data are mean and s.e.m. SCH – SCH 23390. SKF – SKF 81297.
**Figure S4. Dopamine activity negatively correlate with choice and collection latencies. A.** Schematic representation of initiation phase. After initiation rat moves towards touchscreen for the next choice. **B.** Mean dopamine signal after initiation (n = 5). Yellow area assigns the period used for signal quantification. **C.** Choice latency, time between initiation and choice, was affected by the previous trial outcome, as after win rat were slower to make next choice (F_1,5_= 14.535, p = 0.01; Win vs Safe, p = 0.032; Win vs Loss, p = 0.04). **D.** Correlation between dopamine activity (area under the curve) after initiation and choice latency (r = −0.744, p = 0.001). **E.** Schematic representation of choice phase. During choice phase rat approached the screen, touched the screen and moved towards reward collection. **F.** Mean dopamine signal during choice phase (n = 5). Yellow area assigns the period used for signal quantification. **G.** Collection latency differentiated based on the reward outcome. After win animals were the fastest to collect reward, while after loss rats took longer to collect the reward (F_2,10_ = 41.865, p < 0.001; Win vs Safe, p = 0.005; Win vs Loss, p = 0.002; Safe vs Loss, p = 0.012). **H.** Correlation between dopamine activity (area under the curve, AUC) during reward collection and collection latency (r = −0.72, p = 0.002).

## Figures and Tables

**Table 1.** Summary details of drugs used in study.

Drug description	Drug name	Doses (mg/kg)	Dissolving vehicle
dopamine D_1_ antagonist	SCH 23390	0.003, 0.007	0.9% saline
dopamine D_2_ agonist	SKF 81297	0.1, 0.2, 0.5	0.9% saline
α_2A_ adrenergic antagonist	Yohimbine	1, 3, 6	50% sterile water in 0.9% saline
(α_2A_ adrenergic agonist	Guanfacine	0.1, 0.2, 0.5	0.9% saline
GABA_A_ inverse agonist (pharmacological stressor)	FG 7142	1, 4	5-10% DMSO in 0.9% saline with 10-30% HBC

DMSO, dimethyl sulfoxide; HBC, 2-hydroxypropyl-β-cyclodextrin,
