# Noradrenergic modulation of stress induced catecholamine release: Opposing influence of FG7142 and yohimbine

**DOI:** 10.1101/2024.05.09.593389

**Published:** 2024-06-28

**Authors:** Vladimir Visocky, Carleigh J. Turner, Matthew H Lowrie, Anthony Alibro, Fany Messanvi, Yogita Chudasama

**Affiliations:** Section on Behavioral Neuroscience, National Institute of Mental Health, National Institutes of Health, Bethesda, MD 20892, USA

**Keywords:** risky behavior, decision-making, dopamine, loss aversion, amygdala, nucleus accumbens

## Abstract

**Background:**

Life stress modulates decision making, particularly in the face of risk, in some cases prompting vulnerable populations to make suboptimal, life-altering choices. In the brain, stress is known to alter the extracellular release of catecholamines in structures such as basolateral amygdala (BLA) and nucleus accumbens (NAc), but the relationship between catecholamines and decision-making behavior under stress has not been systemically explored.

**Methods:**

We developed an operant touchscreen decision-making task for rats comprising elements of loss aversion and risk seeking behavior. Rats were first injected systemically with an adrenergic ${\text{α}}_{2\text{A}}$-receptor agonist (guanfacine) and antagonist (yohimbine), as well as a partial inverse GABA_A_ agonist, FG 7142, known to induce anxiety and stress related physiological responses in a variety of species, including humans. We then used fiber photometry to monitor NE in the basolateral amygdala (BLA), and DA activity in the nucleus accumbens (NAc) while animals engaged in decision-making and following systemic injections of FG 7142 and yohimbine.

**Results:**

Neither yohimbine nor guanfacine had any impact on decision making strategy but altered motivational state with yohimbine making the animal almost insensitive to the reward outcome. The pharmacological induction of stress with FG 7142 biased the rats’ decisions towards safety, but this bias shifted toward risk when co-treated with yohimbine. In the BLA and NAc, the FG 7142 altered catecholamine release, with systemic yohimbine producing opposing effects on NE and DA release.

**Conclusions:**

Stress induced changes in catecholamine release in the BLA and NAc can directly influence loss sensitivity, decisions and motivation, which can be modulated by the ${\text{α}}_{2\text{A}}$ adrenoreceptor antagonist, yohimbine.

## INTRODUCTION

Everyday decisions involve inherent uncertainty or insufficient knowledge to make informed choice [1]. Such decisions often allow for choices that vary in their ‘risk,’ with one option being relatively safe and another that offer an uncertain potential for major gains and losses. In animals, as in humans, the decision-making is naturally biased towards risk aversion, a default behavioral mode observed in species as diverse as fish [2, 3], birds [4, 5] and bumblebees [6, 7]. Risk aversion is thought to be driven by the affective consequence of loss, also called ‘loss aversion,’ where a given amount of loss impacts humans and other animals more severely than experiencing an equivalent gain [8]. In humans, loss aversion is sensitive to stress [9–11], and patients with neurological or psychiatric illnesses are particularly vulnerable to the detrimental effects of stress thought to cause suboptimal or life-altering choices with long-term negative consequences [12–14]. Here we investigate the effect of stress-induced neuromodulation on risky decision making and brain regions that modulate loss aversion in rats.

Many acute stressors increase extracellular concentrations of norepinephrine (NE) and dopamine (DA) in a number of brain regions, indicating that stress can elicit widespread activation of catecholaminergic neurons [15]. Stress-susceptible brain regions that exhibit structural and/or functional alterations include the amygdala and nucleus accumbens [16–18], both of which contribute significantly to decisions that involve aligning reward gain with loss-sensitivity [19–23]. Stressful stimuli increase NE levels in the basolateral amygdala [24, 25] which produces inhibitory effects through α_2_ receptors to promote the stress response [16, 26, 27]. Thus, NE-modulation of stress in the amygdala could have a role in modulating loss aversion during decision making through stimulation of α_2_ receptors [19, 28]. Midbrain neurons containing and secreting dopamine (DA) are also activated during stress [29–31] causing a change in motivational state mediated by DA in the nucleus accumbens [32]. Consequently, stressed rats reduce their responding for food rewards [33, 34] and bias their decisions to low reward options due to an abnormal lack of motivation or anergia [33, 35]. Thus, both NE and DA can influence decisions under stress by affecting the decision strategy, motivation, and/or sensitivity to reward.

In the present study, we approach this topic with both pharmacology and fiber photometric analysis to elucidate the impact of stress, DA and NE on brain activity during decision making behavior. We induced stress pharmacologically with FG7142, a partial inverse GABA_A_ agonist known to induce anxiety-related behavioral and physiological responses in a variety of species, including humans [36]. In a risky decision-making task, we show that a stress-induced bias towards safety shifted towards indifference by systemic administration of the ${\text{α}}_{2\text{A}}$ receptor antagonist, yohimbine. We further studied the temporal dynamics of NE and DA release related to aspects of trial outcome in the basolateral amygdala (BLA) and nucleus accumbens (NAc), respectively. In both structures, we found that pharmacologically induced stress modulated catecholamine release and that yohimbine led to opposing modulation.

## MATERIALS AND METHODS

Full details of materials and methods is provided in supplemental Fig. S1. All experimental procedures were approved by NIMH Institutional Animal Care and Use Committee, in accordance with the NIH guidelines for the use of animals.

### Subjects

Male Long-Evans rats (Inotiv, Indianapolis, IN, USA) were pair-housed in a temperature-controlled room (23.3 °C) under diurnal conditions (12:12 h light: dark). Rats were maintained at 90% of the free-feeding weight and water was available for at least 2hrs a day.

### Decision making behavior

**Figure 1.**
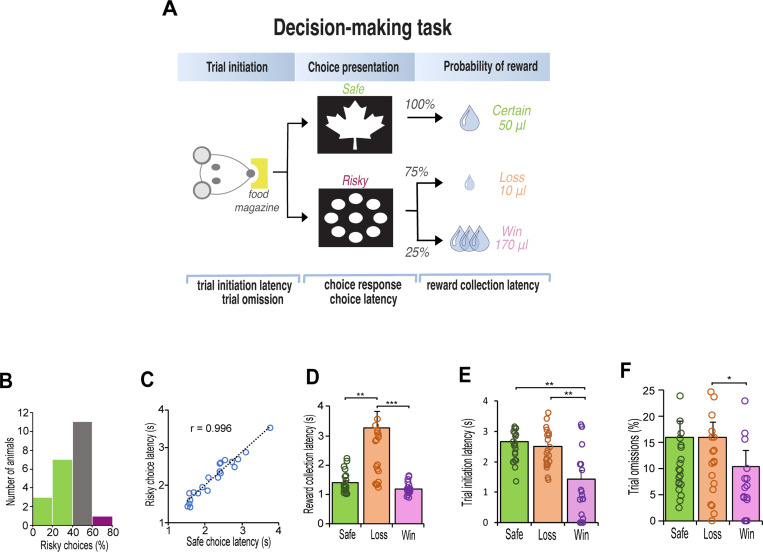
Schematic illustration of decision-making task and baseline behavior after 15 days of training. **A.** Rats initiated each trial with a nosepoke before choosing the ‘safe’ image (leaf) or the ‘risky’ image (circles). The safe image delivered a 50-μl reward. The risky image delivered a 10μl reward with 75% probability and a 170μl reward with 25% probability. The expected value of each choice was 50 μl of sucrose. **B**. Histogram of risk preference. Green indicates risk-averse rats (> 40% risky choices); grey indicates indifferent rats (> 40% and < 60%); purple indicates risk-seeking rats (> 60%). **C.** Latency to choose the safe or risky option were equal. **D**. Reward collection latency was slower for reward loss reward (10-μl sucrose) for all rats (n = 22, Friedman test, $\chi^{2}=42.91$, p < 0.001; Safe vs Loss, Adj. p = 0.002; Win vs Loss, Adj p < 0.001). **E**. Latency to initiate a trial was always faster after a win (170μl sucrose) relative to after a safe/certain reward or reward loss (n = 22, latency x reward outcome interaction F_1,22_ = 16.913, p > 0.001; Safe vs Loss, p = 0.001; Win vs Loss, p = 0.002). **F**. After losing a reward, rats were more likely to omit next trial than after winning a high reward (n = 22, Friedman test, $\chi^{2}=7.747$, p = 0.021; Win vs Loss, p = 0.031). Data are mean and S.E.M. *** p < 0.001, ** p < 0.01, * p < 0.05.

**Figure 2.**
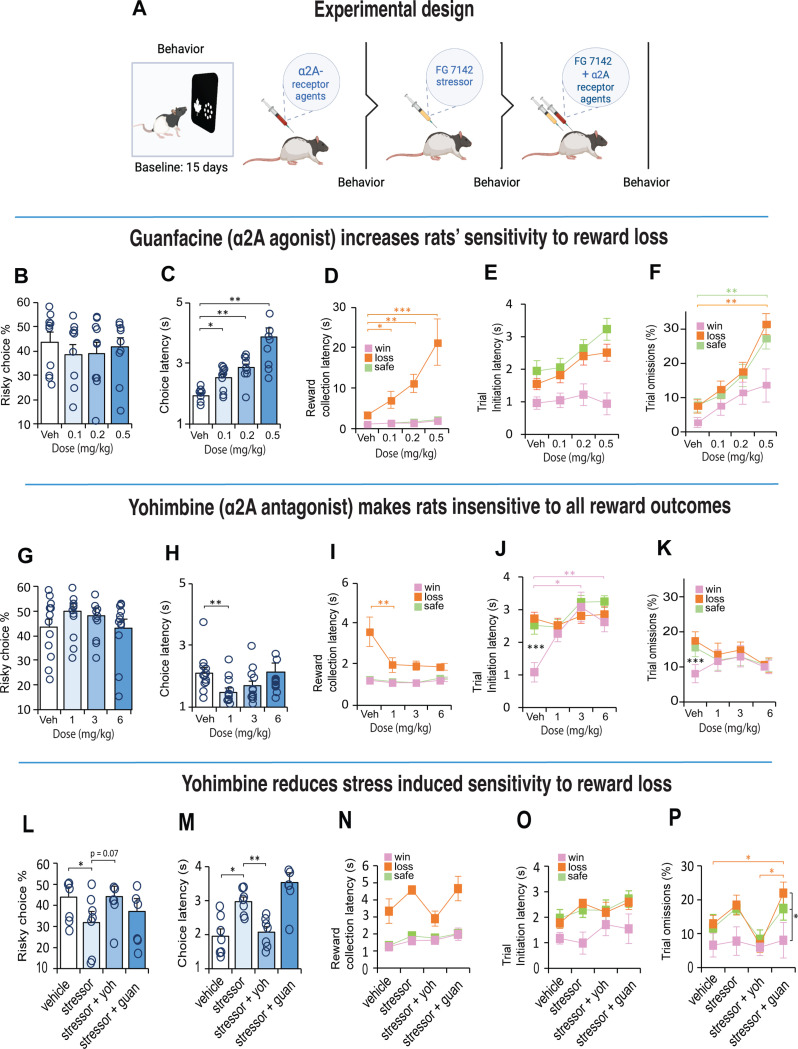
The effect of systemic ${\text{α}}_{2\text{A}}$ agonist and antagonists on decision making behavior. **A.** After 15 days of training, rats (n = 22) received a counterbalanced dose of an α2A agonist (guanfacine) and antagonist (yohimbine) 30 minutes before the test. Two weeks later, rats were injected with FG7142 to induce stress and tested again 30 minutes later for their decision making behavior. Subsequently, the animals were co-injected with the stressor and α2A receptor specific drug before behavioral testing. **B, G**. Risky choices are not affected by systemic ${\text{α}}_{2\text{A}}$ drug treatments. **C, H.** Guanfacine slowed down decision speed (Guanfacine, n =10, latency x dose interaction F_3,27_ = 24.512, p = 0.004; Veh vs all doses p < 0.05). In contrast, yohimbine made rats faster in their response speed (n = 12, Friedman test, $\chi^{2}=15.5$, p = 0.001; Veh vs Low, p = 0.01). **D, I.** Reward collection latencies increased with guanfacine (Guanfacine, n = 10, latency x dose interaction F_3,23_ = 14,41, p < 0.001; Veh vs all doses p < 0.05). Yohimbine made rats faster after loss (yohimbine, n = 10, latency x dose interaction F_2,17_ = 9.85, p = 0.002; Veh vs Low dose, p = 0.005). **E, J.** Trial initiation latency was generally faster after winning a high reward when injected with guanfacine, but not with yohimbine (Yohimbine, n = 12, latency x dose interaction F_3,33_ = 4.84, p < 0.001; Vehicle, Win vs Loss, p < 0.001). Yohimbine Low dose x latency interaction F_2,22_ = 0.457, p > 0.05. Low – low dose. Med – medium dose. High – high dose. Data are mean and S.E.M. *** p < 0.001, ** p < 0.01, * p < 0.05. **L-P.** Pharmacological stress induced by an i.p. injection of 4 mg/kg of FG 7142 reduced risky choices and increased their choice latency, while co-treatment of FG 7142 with yohimbine produced opposing effects; rats were faster in their choices and increased their choice of risky options to a level of indifference. Combined treatment of FG 7142 with guanfacine made rats slow and omit more trials after a reward loss.

Rats were tested on a decision making task using the operant touchscreen platform. Rats chose between two different stimuli presented on the left and right side of the touchscreen monitor (Fig. 1A). Nosepoke touches to the ‘safe’ stimulus (leaf) always resulted in the delivery of the small 50μl sucrose reward. Responses to the ‘risky’ stimulus (circles), delivered a small 10μl sucrose reward 75% of the time, or a large 170μl sucrose reward 25% of the time. Following stable baseline performance, rats (n=12) were treated IP with an adrenergic ${\text{α}}_{2\text{A}}$-receptor agonist (guanfacine) and antagonist (yohimbine). Two weeks later, we induced stress with a pharmacological stressor, FG 7142, a GABA_A_ inverse agonist (Fig 2A) which induces biochemical changes that mimic stress or anxiety similar to those elicited by mild aversive conditioning [37, 38], and therefore occurs independently of nociceptive stimuli. We then sought to alter the effects of the stressor by co-injecting it with the noradrenergic receptor specific drug.

### Fiber photometry

In a subset of pretrained animals (n = 9), we injected, unilaterally, a genetically encoded fluorescent NE sensor, GRAB_NE_ (pAAV-hSyn-GRAB_NE1m) in the basolateral amygdala (BLA), or a DA sensor, dLight in the nucleus accumbens (NAc) to monitor NE and DA neural activity while animals engaged in the decision-making task. Subsequently, we examined NE and DA responses following systemic injections of vehicle, yohimbine (1mg/kg) and FG7142 (4mg/kg).

### Statistical analysis

The behavioral data were processed using custom-written programs in R and analyzed using SPSS Statistics 25.0 (IBM, Chicago, IL, USA). Incomplete data with sessions comprising less than 30% of free choices trials were not used for statistical analysis. All data were tested for normality and transformed accordingly before statistical significance testing (see Fig. S1 for full details).

## RESULTS

### High sensitivity to loss impacts motivational state

After 3 weeks of testing, rats displayed consistent choice preferences across three consecutive days (${\chi^{2}}_{\left(2\right)}=0.302$, p = 0.86). Similar to human choice behavior [40], the rats were either indifferent in their choices (n=11) or exhibited stable risk-aversion (n=10). Only one rat exhibited risk taking behavior by choosing the risky option more than 60% of the time (Fig. 1B). Regardless of their choice, the speed at which the animals made their response did not differ between the safe or risk option (t_(21)_ = 0.97, p = 0.34), and correlated strongly (Pearson r_(21)_ = .97, p < 0.001) such that the latency to choose the safe or risky option were equal (Fig 1C). However, their motivation to collect reward was highly influenced by their outcome, especially following selection of a risky option that led to a loss (${\chi^{2}}_{\left(2\right)}=42.09$, p < 0.001). Posthoc tests confirmed that the latency to collect reward was twice as fast following delivery of the safe-certain reward (Z = 3.47, p = 0.002) and a risk-win reward (Z = 6.48, p < 0.001) relative to a risk-loss (Fig. 1D). Their motivation to initiate the next trial was also influenced by the outcome of the choice; after choosing the risky option that led to a reward loss, the speed to initiate the next trial was substantially slower than following a win (F_(1, 23)_ = 16.91, p < 0.001, Fig. 1E), and in many cases resulted in trial omissions (Z = 2.56, p = 0.031, Fig. 1F).

### ${\text{α}}_{2\text{A}}$ – adrenoceptors modulate sensitivity associated with reward loss

We next examined how choice for safe or risky options were modulated by noradrenergic drugs that acted on the ${\text{α}}_{2\text{A}}$-receptor. There is some evidence that stimulation of ${\text{α}}_{2\text{A}}$-receptors affects some forms of decision making even in normal animals [41, 42]. To test this possibility, we first injected a cohort of trained rats with low, medium, and high doses (Fig. S1) of guanfacine (${\text{α}}_{2\text{A}}$-receptor agonist) and yohimbine (${\text{α}}_{2\text{A}}$-receptor antagonist), in separate sessions, each counterbalanced with vehicle (Fig. 2A). Neither drug had any impact on the animals’ preference for the choice at any dose (guanfacine: ${\chi^{2}}_{\left(3\right)}=4.09$, p = 0.252; yohimbine: ${\chi^{2}}_{\left(3\right)}=5.5$, p = 0.139; Fig. 2B, G), but substantially altered the animals’ sensitivity to loss and motivational state, in opposite directions. In general, guanfacine demotivated the animals by slowing them down without impacting their motoric abilities. For example, their latencies to make a choice increased with higher doses (F_(3, 27)_ = 24.512, p = 0.001; Fig. 2C), but their latencies to collect reward depended on the reward outcome (F_(3, 23)_ = 14.41, p < 0.001). When the animal chose the risky option and experienced a reward loss, these animals were disproportionally slower in collecting the reward which got worse with increasing dose (F_(3, 27)_ = 23.811, p < 0.001; Fig. 2D). The same animals, however, were distinctly fast to collect reward following a choice response that led to a win or a safe-certain reward at all doses. Thus, the long latencies could not be explained by mere sedation, but a specific sensitivity to reward loss. Similarly, the latency to initiate a trial following the different reward outcomes was impacted across all doses (F_(3, 25)_ = 2.93, p = 0.056; Fig. 2E) as were the number of omission ( F_(6, 54)_ = 4.06, p = 0.002; Fig, 2F).

Opposite to the effects of guanfacine, antagonizing the ${\text{α}}_{2\text{A}}$-receptors with yohimbine made the animals almost insensitive to the reward outcomes; their choice strategy remained constant (Fig. 2G), they were faster to make a choice response especially at the low dose (${\chi^{2}}_{\left(3\right)}=15.5$, p = 0.001; Fig. 2H) and comparatively faster than guanfacine (compare with Fig. 2C). A small difference emerged between reward loss and reward win collection latencies (F_(2,18)_ = 7.75, p = 0.005), but relative to vehicle, the yohimbine made rats substantially faster to collect rewards even when the choice led to a large reward loss (Fig. 2I). Moreover, Fig. 2J shows that with yohimbine, the animals were so insensitive to the different reward values that their motivation to initiate the next trial was identical for all reward outcomes including after reward losses (F_(2, 22)_ = 0.46, p = 0.64), but not with vehicle (F_(2, 22)_ = 24.4, p < 0.001). A similar pattern was observed for omissions (Vehicle: F_(2, 22)_ = 31.6, p < 0.001; yohimbine: F_(2, 22)_ = 1.33, p = 0.29; Fig. 2K)

Since many psychiatric disorders characterized by risky decision making are associated with dysregulated dopamine transmission [43, 44], in a separate cohort of rats, we also examined the effects SKF 81297 (dopamine D_1_ receptor agonist) and SCH 23390 (dopamine D_1_ receptor antagonist) for comparison. The D_1_ antagonist, while not affecting the animal’s choice behavior affected the animals motivation by increasing their latencies following a reward loss, but there was no major impact on decision making behavior with the D_1_ agonist (Fig. S2).

### Yohimbine reduces stress-induced sensitivity to reward loss

We next examined if decision-making was sensitive to physiological stress. Due to the habituation caused by repeated exposure to stress, we gave rats a systemic 4mg/kg dose of a pharmacological stressor known as FG 7142 (henceforth known as stressor or FG stressor; Fig 2A). This drug is known to mimic the effects of uncontrollable stress linked to anxiety [45] and increases catecholamine turnover in various limbic associated areas including the BLA and NAc [46–48] thought to influence choice behavior in humans and animals [49]. The stressor veered rats’ choices towards safety (F_(3, 18)_ = 6.408, p = 0.004; veh vs stressor, p = 0.016; Fig. 2L) and made them slower in their response (F_(3, 18)_ = 25.859, p < 0.001; veh vs stressor, p = 0.026; Fig. 2M) suggesting they were less motivated to engage in risk taking behavior. Apart from an increased sensitivity to reward loss, other aspects of motivation were relatively intact (Figs. 2N-P). In humans, however, the co-administration of the stress hormone hydrocortisone with yohimbine diminishes loss aversion [11]. To test this possibility, we co-injected the FG stressor with yohimbine and found, that their concurrent actions tended toward reduced loss aversion in rats relative to the stressor alone (F_(3, 18)_ = 6.408, p = 0.004; FG vs FG + Yoh, p = 0.07; Fig. 2L), and made them noticeably faster in their choices (p = 0.008; Fig. 2M). It also increased their motivation to collect low rewards, initiate trials and reduced the number of omissions after a reward loss (Figs 2N-P). In contrast, the behavior associated with combined guanfacine, and the FG stressor was equivalent to the stressor alone (all p>0.05, NS).

### FG stressor and yohimbine produce opposing effects on BLA-NE release

**Figure 3.**
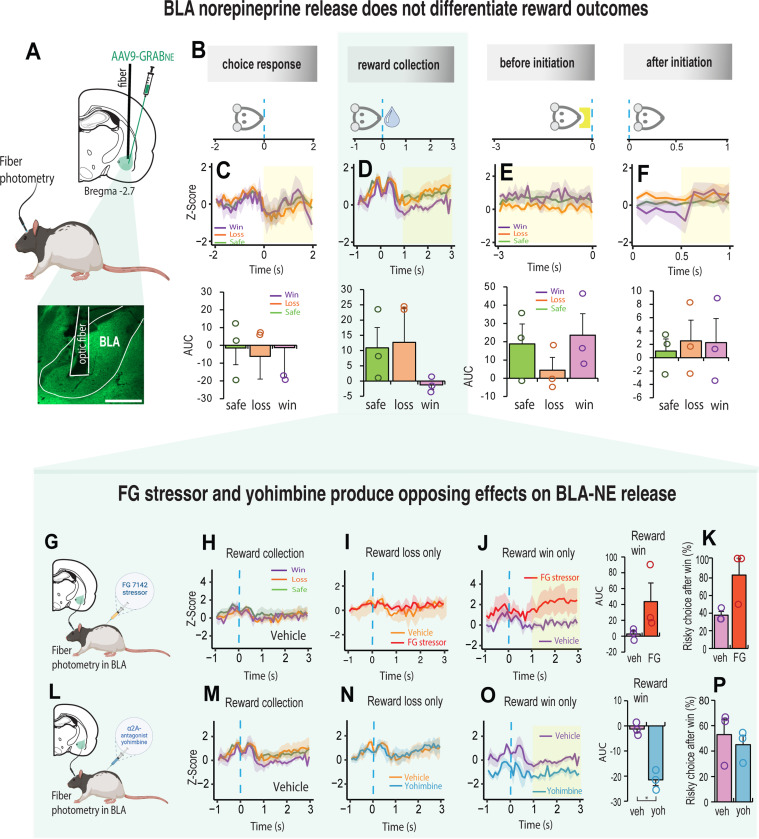
FG stressor and yohimbine produce opposing effects on BLA-NE release **A.** Schematic illustration of AVV9-GRAB_NE1m expression in the BLA and histology showing optic fiber placement above the injection site. **B.** Schematic illustration of how NE signal was aligned to four specific events in the trial. Choice response: the rat touched the screen and moved towards reward collection. Reward collection: rat entered the food magazine to collect the reward. Before initiation: 3 second before the next trial was initiated. After initiation: 1 second after trial initiation, during which rat moves towards touchscreen for the next choice. **C-F** Top row. Mean noradrenaline signal during choice, reward collection, after initiation and before initiation (n = 3). The dynamics of NE release are different for reward wins (purple), safe/certain rewards (green) and reward loss (orange). The yellow shaded area assigns the period used for the area under the curve analysis (AUC). **C-F** Bottom row. Shaded yellow area assigns the period for the area under the curve analysis (AUC). **G.** Schematic illustration of fiber photometry probe in BLA combined with FG7142 injection. **H.** NE signal in the BLA aligned to reward collection for vehicle (n = 3). **I.** NE signal in BLA following vehicle (orange) and FG7142 (red) treatment are indistinguishable reward collection resulted in reward loss. **J.** FG stress (red) induced NE signal for collection of high reward win increased relative to vehicle (purple). **K.** The increase in NE signal after collecting a high reward was associated with a higher propensity to make risky choices. **L.** Schematic illustration of fiber photometry probe in BLA combined with yohimbine injection. **M.** NE signal in the BLA aligned to reward collection with vehicle. NE signal did not differentiate between win (purple), safe (green) and loss (orange) reward outcomes. **N.** NE signal for reward collection after a reward loss was indistinguishable between vehicle (orange) and yohimbine (blue). **O.** NE signal in BLA for collection of a high reward win in yohimbine (blue) treated rats reduced relative to vehicle (purple). **P.** The reduction in NE signal did not influence the subsequent choice. Data are mean and S.E.M. AUC – area under the curve. Veh – vehicle. FG – FG7142. Yoh – yohimbine. Scale bar 500 μm. ** p < 0.01, * p < 0.05.

We next asked if the fluorogenic NE reporter GRAB_NE,_ could capture the rapid dynamic properties of NE release for decisions that led to different reward outcomes. We focused on the basolateral amygdala (BLA) because it is diffusely innervated by NE projecting neurons from the locus coeruleus [50–52], and NE levels in the BLA increase with presentation of stressful stimuli [24, 25]. We injected rats with an AAV that expressed GRAB_NE_ in the BLA and implanted an optic fiber above the injection site (Figs. 3A, S3A). After a minimum of 4 weeks to allow the expression of the viral sensor, rats were placed in the test chambers and assessed on their decision-making while recording the GRAB_NE_ fluorescence. The fluorescent signal was aligned to four specific events in the trial: the choice response, reward collection, before trial initiation and after trial initiation (Fig. 3B). Changes in NE release were not observed in the BLA during the decision itself such that the kinetics of the NE response were relatively equivalent before and after the choice (Fig. 3C). A double activation of signal for NE release in the BLA was observed just before and immediately after reward collection regardless of the trial outcome (Figs. 3D, S4A-C).

Subsequently the signal declined especially for choices that led to large reward wins. The signal for the remaining trial events did not differentiate the reward outcomes (Figs. 3E, F). Thus, NE release in the BLA does not correlate directly with choices influenced by risk or uncertainty. However, since yohimbine and the FG stressor altered the animal’s sensitivity to reward outcome when injected systemically (Figs. 2L-P), we measured NE signal in the BLA following the injection of each of these agents (Figs. 3G, L). We found these drugs to have opposing effects on NE release but only when we aligned the signal to reward collection (Figs. 3G-P). While the stressor had no impact on the NE response for reward losses (Fig. 3I), it increased the NE signal following a large reward win (Fig. 3J; t_2_ = 2.02, p = 0.181). Although it did not affect decision strategy (t_2_ = 2.675, p = 0.11), there was a higher propensity to make risky choices after a reward win Fig. 3K). Conversely, systemic yohimbine reduced NE release in the BLA relative to vehicle when the choice led to a large reward win (t_2_ = 9.407, p = 0.011) but this did not influence the subsequent choice (Fig. 3L-P). The BLA-NE signal did not change for either drug when the reward collection followed a loss (Fig. 3I, N). These results suggest that ${\text{α}}_{2\text{A}}$ – adrenoceptors can potentially suppress or shift the effects of stress induced risky behavior by making the animal choose safer options.

### Stress induced DA response in the NAc is modulated by ${\text{α}}_{2\text{A}}$ – adrenoceptors

**Figure 4.**
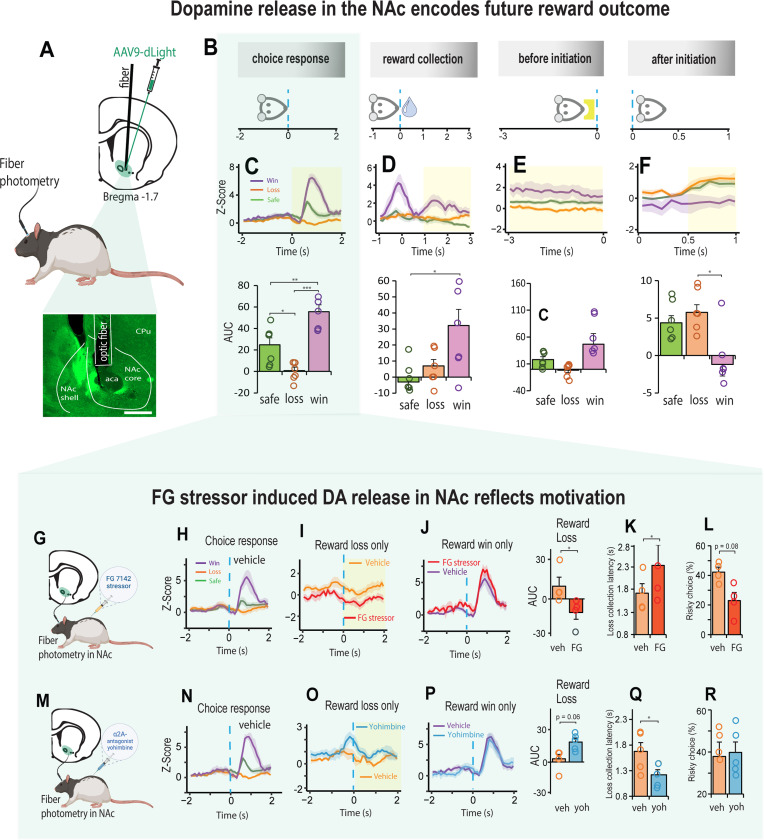
Stress induced DA release in the NAc reflects motivation during decision-making. **A** Schematic representation of an AVV9-dLight expression in the NAc and histology showing optic fiber placement above the injection site. **B**. Schematic illustrating how NE signal was aligned to four specific events in the trial. Choice response: the rat touched the screen and moved towards reward collection. Reward collection: rat entered the food magazine to collect the reward. Before initiation: 3 second before the next trial was initiated. After initiation: 1 second after trial initiation, during which rat moves towards touchscreen for the next choice. **C - F.** DA signal in NAc during the four trial events shown in B (n = 5). **C.** Strength of DA release aligned to the choice response varied systemically as a function of expected reward value such that DA release was high when there was an increase in future reward value (a reward win: purple), inhibited when there was decrease in future reward value (a reward loss: orange), and intermediate when the future reward was low but certain (green). Win vs Loss, p < 0.001; Win vs Safe, p = 0.002; Loss vs Safe, p = 0.017). Shaded yellow area assigns the period for the area under the curve analysis (AUC). **D.** The dynamics of the DA signal was greater after collection of a large reward win than after safe/loss reward collection. Win vs Loss, p = 0.097; Win vs Safe, p = 0.029). **E.** Before initiating the next trial, the DA signal was slightly elevated if the previous choice was a win. **F.** The DA signal lowered substantially once the next trial was initiated if the previous choice was a win. Win vs Loss, p = 0.024; Win vs Safe, p = 0.054). **G.** Schematic representation of dopamine signal collection in NAc combined with FG7142 injection. **H.** DA signal during aligned to choice response (n = 4). **I.** Reduced DA signal when choice response led to a reward loss for Veh (orange) and FG stressor (red). **J.** Elevated DA signal for choice response leading to future reward win with stressor (red) and vehicle (purple) were similar. **K-L.** Reduced DA signal with stressor injection leading to reward loss was associated with long reward collection latencies and an increase in choices towards safety. **M.** Schematic representation of DA signal in NAc combined with yohimbine injection. **N.** DA signal aligned to the choice phase following Veh injection (n = 5). **O.** DA signal when choice response led to reward loss with yohimbine (blue) was higher relative to vehicle (orange). **P.** DA signal when choice response led to reward win following vehicle (purple) and yohimbine (blue) were indistinguishable. **Q-R.** Decrease in DA signal following reward loss was associated with faster reward collection latencies but did not affect the animal decision strategy (loss collection latency, t_4_ = 2.783, p = 0.049). Data are mean and S.E.M. AUC – area under the curve. Veh – vehicle. FG – FG7142. Yoh – yohimbine. Scale bar 500 μm. *** p < 0.001, ** p < 0.01, * p < 0.05.

We also asked if stress induced changes in motivation could be differentially modulated by dopamine (DA). There is much evidence however, that stress has profound effects on the mesoaccumbens dopamine system [30, 31, 53] and that projections from dopaminergic nuclei to the nucleus accumbens (NAc) play an important role in motivated decision-making behavior [54, 55]. Accordingly, we first recorded changes in fluorescence of the genetically encoded dopamine (DA) sensor, dLight expressed in the NAc to examine changes in the DA response during decision-making (Fig. 4A, S3B). The highest peak of DA release in the NAc was observed after the choice was made, and it related to the value of the future reward (Figs. 4B-C, S4D-F). Thus, DA release was high when there was an increase in future reward value (a reward win), inhibited when there was decrease in future reward value (a reward loss), and intermediate when the future reward was low but certain (Choice: F _(2, 10)_ = 57.39, p < 0.001; Fig. 4C). Moreover, the DA response for the winning choice peaked high during reward collection and elevated again *after* reward collection (F _(1, 5)_ = 6.95, p = 0.031; Fig. 4D). High DA release after a win persisted to some degree until the animal initiated the next trial (before initiation: F _(1, 5)_ = 3.3, p = 0.079; Fig. 4E), which could potentially explain the high motivational state characterized by faster trial initiations and reduced omissions (Fig 1D - F). After the next trial was initiated, the DA signal reversed; the previous reward wining outcome resulted in a reduction in DA release whilst the previous low reward outcomes enhanced DA release in the NAc (after initiation: F _(2, 10)_ = 8.06, p = 0.008; Fig. 4F).

We next examined changes in DA release in the NAc following systemic injections of the FG stressor (Fig. 4G-L) and found that it had no impact on the DA signal for choices that led to a reward win, but for those choices that led to a reward loss, the DA signal declined relative to vehicle **(Fig. I-J).** Moreover, as we observed through systemic injections (Fig. 2L), there was an associated increase in loss sensitivity characterized by slower collection of low rewards and increased aversion to risk (AUC, t_3_ = 4.299, p = 0.023; loss collection latency, t_3_ = 3.875, p = 0.03; % risky choice, t_3_ = 2.6, p = 0.08; Fig. 4K, L). We then discovered that the FG stressor and yohimbine had opposing effects on NAc-DA raising the possibility that the sensitivity associated with loss could be potentially reduced with α2A - adrenoceptors (Fig. 4M-R). This not only increased DA release in response to reward loss (Fig. 4O), it increased the rat’s motivation by making them faster to collect a low reward without affecting their decision strategy (Fig. 4Q, R; AUC, t_4_ = 2.55, p = 0.063; loss collection latency, t_4_ = 2.783, p = 0.049).

## DISCUSSION

There were three main findings: 1) pharmacological stress shifted behavior toward safe decisions, and this effect was reduced by yohimbine, 2) following large gains after a risky choice, the level of NE release in the BLA was increased by a pharmacological stressor but decreased by yohimbine, 3) following a loss after a risky choice, the level of DA in the NAc was decreased by a pharmacological stressor but increased by yohimbine. These opposing effects of the FG stressor and yohimbine on catecholamine release in these critical structures during the outcome of risky decisions provides a plausible explanation of why yohimbine shifts choice behavior in the face of a pharmacological stressor. Specifically, it points to the role of ${\text{α}}_{2\text{A}}$ – adrenoceptors as being critical to the remediation of the stress effects.

### Opposing influence of ${\text{α}}_{2\text{A}}$ adrenoceptor activation

One major finding of our study is that NE modulation at ${\text{α}}_{2\text{A}}$ adrenoceptors has a powerful influence on how reward loss is incorporated into the animals’ decision. We found that, similar to the pharmacological stressor, FG 7142, loss aversion was disproportionately high in animals injected with the ${\text{α}}_{2\text{A}}$ agonist, guanfacine; they were slow in making choices, collecting their rewards, and initiating trials, but only when their choice resulted in a low reward outcome. When the choice outcome resulted in a high reward, rats showed normal levels of motivation and speed of response even at the high dose, confirming that the demotivating effects of systemic guanfacine following reward loss were not due to sedative effects of the drug. Guanfacine, through its postsynaptic actions in the prefrontal cortex, is known to enhance cognitive functions that subserve attention and working memory [56, 57]. One possibility is that while guanfacine made the ${\text{α}}_{2\text{A}}$ adrenoceptor highly sensitive to decisions that lead to major losses, it functioned to enhance or focus attention most efficiently towards choice outcomes with positive consequences.

In contrast, when the ${\text{α}}_{2\text{A}}$ receptors were antagonized with yohimbine, these same animals showed exaggerated focus and speed, with no evidence of loss aversion. In fact, in their eagerness to respond, they often failed to discriminate the different reward values associated with the choice outcome. Consequently, these animals worked equally fast for all trials even when their chosen option lead to a loss. While it is possible that the fast, indiscriminate responding with yohimbine was caused by an impulsive-like state [58–60], they were not prone to make riskier choices. In fact, despite the robust alterations in the animals’ motivational state, neither yohimbine nor guanfacine, when systemically administered alone, had any impact on the animal’s decision strategy.

### ${\text{α}}_{2\text{A}}$ adrenoceptor modulation of stress induced catecholamine release

Although systemic injections of the FG stressor shifted rats’ preferences for low-risk choices, its impact on NE activity in the BLA was markedly different. The photometry trace revealed an elevated NE signal in BLA neurons for the large reward win, which further inclined the rat to make high-risk choices. The high NE signal is consistent with the general finding that stressful stimuli increase NE release in the BLA [24, 48, 61]. In fact, when rats were not stressed, there was very little variation in NE release in the BLA suggesting that basal levels of BLA-NE have little impact on decision-making under risk. Notably, the NE signal did not change when choices resulted in a reward loss suggesting that NE in the BLA, while sensitive to emotionally stressful or fearful contexts [62, 63], may not encode the affective consequence of loss, or ‘loss aversion.’ This difference may be due to the nature of the stressor or its intensity which differentially impacts the activity of noradrenergic neurons in different brain regions [64–66]. Since benzodiazepine receptor binding is altered by stress [67, 68], and NE acts on GABAergic cell populations indigenous to the BLA region [69, 70], the inclination to be risky may be related to impaired NE modulation of GABA transmission in the BLA. Our finding that systemic injections of yohimbine reduced the BLA-NE signal without affecting decision strategy is consistent with this hypothesis.

We note that unlike other published reports [71, 72], the effects of the DA D_1_ agonist/antagonist on decision making were modest at best (Fig. S2), but not uncommon [73–75]. In our case, the DA D_1_ agonist and antagonist had a noticeable effect on motivation which was further supported with the photometric analysis of DA release in the NAc. In the absence of stress, the DA signal in the NAc encoded both, the reward value associated with the choice, as well as the magnitude of the high reward which was sustained for some time after reward collection. Notably, the size of the NAc-DA signal positively correlated with the rat’s motivation (Fig. S5), a finding contrary to that of Eshel et al., [54] where motivation, characterized by the willingness to overcome the cost of working in mice, was found to be negatively correlated with NAc-DA activity. In the present study, the sensitivity to reward loss exacerbated when the animal was stressed with FG 7142. This resulted in a reduced DA signal in the NAc, whereas yohimbine increased the DA signal. In fact, with yohimbine, the NAc-DA signal was high even *before* the choice was made, and this predicted the animals increased motivation to decide faster on their options and collect their rewards, even for low rewards. Thus, although DA activity in the NAc is sensitive to experienced loss during stress, we found that the reduced motivation exhibited by these animals for low reward choices can be potentially countered with an ${\text{α}}_{2\text{A}}$ adrenoreceptor interaction with GABA_A_ receptors in the NAc.

### Concluding remarks

We applied methods of behavior, psychopharmacology and fiber photometry to measure the neural dynamics of stress modulators in brain regions that affect decision-making in rats. Like humans, we showed that rats imitate the loss aversion effect and its associated outcome-based motivations. Our results support the proposal that stress induced changes in catecholamine release in the BLA and NAc can directly influence loss sensitivity, choices and motivation, which can be modulated by the ${\text{α}}_{2\text{A}}$ adrenoreceptor antagonist, yohimbine. Stress associated catecholamine release exacerbates vulnerability to a variety of clinical conditions in which patients engage in decision making involving risks and rewards. Elucidating the complex interplay between neuromodulatory circuits that mediate this form of decision making will improve our understanding of the dysfunctional pathways linking the stress response to suboptimal life-altering choices.

## Supplementary Material

Supplement 1
