# Supplementary material for: Noradrenergic modulation of stress induced catecholamine release: Opposing influence of FG7142 and yohimbine
Source: bioRxiv. 2024 May 25:2024.05.09.593389. Originally published 2024 May 9. Preprint. [Version 2] doi: 10.1101/2024.05.09.593389 (PMC11100835; doi:10.1101/2024.05.09.593389)

bioRxiv preprint doi: https://doi.org/10.1101/2024.05.09.593389; this version posted May 25, 2024. The copyright holder for this preprint (which was not certified by peer review) is the author/funder. This article is a US Government work. It is not subject to copyright under 17 USC 105 and is also made available for use under a CC0 license.

## Supplemental Information

# Noradrenergic modulation of stress induced catecholamine release: Opposing influence of FG7142 and yohimbine.

Vladimir Visocky PhD, Carleigh J. Turner MA, Matthew H Lowrie BS, Anthony Alibro BA, Fany Messanvi PhD, Yogita Chudasama PhD

Section on Behavioral Neuroscience, National Institute of Mental Health, National Institutes of Health, Bethesda, MD 20892, USA

**Address for correspondence:**
Dr. Yogita Chudasama
National Institute of Mental Health
Building 35A, Room 2D-804
Bethesda, MD 20892
Tel: 301 594-4569
Email: Yogita.chudasama@nih.gov

bioRxiv preprint doi: https://doi.org/10.1101/2024.05.09.593389; this version posted May 25, 2024. The copyright holder for this preprint (which was not certified by peer review) is the author/funder. This article is a US Government work. It is not subject to copyright under 17 USC 105 and is also made available for use under a CC0 license.

## Figure S1: Materials and Methods

### Subjects

Male Long-Evans rats (Inotiv, Indianapolis, IN, USA) weighing 250-280g at the start of behavioral training were used for these studies. They were pair-housed in a temperature-controlled room (23.3 °C) under diurnal conditions (12:12 h light: dark). All testing occurred at a regular time during the light period. Rats were maintained at 90% of the free-feeding weight and water was available for at least 2hrs a day. All experimental procedures were approved by NIMH Institutional Animal Care and Use Committee, in accordance with the NIH guidelines for the use of animals.

### Decision-making behavior

*Touchscreen Operant Platform.* Behavioral testing was in eight automated touchscreen operant chambers (Lafayette Instrument Company, Lafayette, IN, USA) each comprising a standard operant chamber fitted with a touchscreen. Each chamber was individually housed in a sound-attenuating cabinet and illuminated by a 3W houselight mounted on the ceiling of the cabinet. Computer graphic stimuli composed of white geometric symbols on a black background were presented on a touch-sensitive monitor (9” W × 10” H). A black mask made of anodized aluminum was attached to the face of the screen approximately 1.5cm from the surface of the display. The mask served to restrict the rats access to the screen except through two response windows (3” W × 3” H). Opposite the touchscreen was a precision liquid food pump (Lafayette Instrument Company, Lafayette, IN, USA) which delivered 10% sucrose solution into a food magazine. A light-emitting diode illuminated the food magazine. Magazine entries were detected by photocells located at the entrance of the food magazine. The apparatus and online data collection for each chamber were controlled with ABET II Software for operant control (Lafayette Instrument Company, Lafayette, IN, USA) interfaced with the Whisker control system for research (Cardinal and Aitken, 2010).

*Pretraining.* Following habituation to the testing chamber, rats were trained to enter the food magazine to collect to 50 µl sucrose reward. When rats were able to make 50 food magazine entries and thereby retrieve 50 rewards, they were trained to retrieve 50 rewards by touching one of two illuminated white squares (3”x 3”) presented on the touchscreen. Next, the rats were trained to initiate a trial by making a nose poke entry into an illuminated food magazine which resulted in the presentation of two white squares. In the first phase, a nosepoke touch to either of the white squares led to sucrose delivery. In the second phase, only one square was presented on either the left or the right side of the screen. A minimum of 50 nosepoke touch responses were required for each phase. Each pretraining session lasted 30 mins. In the last phase, the nose poke to receptacle initiated a white square on one of the randomized sides. To pass the training, rats were expected to touch each side more than 50 times. Once all 4 training phases were completed, rats were exposed to the behavioral task described in Fig. 1A. On average, rats were pretrained for ~ 5 days.

bioRxiv preprint doi: https://doi.org/10.1101/2024.05.09.593389; this version posted May 25, 2024. The copyright holder for this preprint (which was not certified by peer review) is the author/funder. This article is a US Government work. It is not subject to copyright under 17 USC 105 and is also made available for use under a CC0 license.

Following habituation to the chamber, rats were trained to reliably initiate trials, touch the screen and collect 10% sucrose solution as a reward.

*Behavior.* Rats chose between two different computer graphic stimuli presented on the left and right side of the touchscreen monitor **(Fig. 1A)**. Each stimulus indicated differences in reward size and probability of outcome. Responses to the 'safe' stimulus (leaf) always resulted in the delivery of the small 50µl sucrose reward. Responses to the 'risky' stimulus (circles), delivered a small 10µl sucrose reward 75% of the time, or a large 170µl sucrose reward 25% of the time. The left/right positions of the risky and safe images were pseudorandomly determined thereby eliminating the potential confound of a side bias. Importantly, the expected value of the reward remained the same regardless of the animal's choice. Each session started with 50 forced trials during which the safe or risky stimulus was presented to demonstrate the outcome associated with the stimulus. The remaining 200 trials were free choice trials in which rats could choose between both stimuli.

Each trial was signaled by the illumination of the magazine and house light. A nosepoke entry into the food magazine triggered the presentation of the stimuli for 10 sec. Following a successful response, the stimuli disappeared, all lights extinguished, and the chamber entered an intertrial interval (ITI) state of 10 sec. The next trial was signaled by the illumination of the food magazine and the houselight. Failure to make a nose poke entry with the 10 sec stimulus duration was recorded as an omission, and the box was returned to the intertrial state. The trial was then repeated until the rat received the full complement of 200 forced choice trials for that session.

Rats reached stable baseline performance in three weeks. Their fraction of risky choices across three consecutive days varied by less than 15 % (mean range = 7 %, and s.d. = 3). Rats that showed persistent side biases were excluded (n=2). The following formula was used to calculate the percentage of risky choices: % Risky = (Number of risky choices (Losses and Wins)/Total number of choices) *100). We also reported: choice response latency (time between trial initiation and topuch response), reward collection latency (time after choice response and collection of reward type (i.e., safe, loss or win), proportion of omission (failure to initiate the next trial after reward collection). The following formula was used to calculate the proportion of omission: (% omissions = (N of omissions after particular reward type)/(N of omissions and responses after particular reward type)

**Systemic pharmacology: Drug preparation and experimental design**

All drugs were administered systemically i.p. and counterbalanced with vehicle. Doses of drugs were calculated as the salt and dissolved in the appropriate vehicle. **Table 1** lists all drugs, dosages, and dissolving vehicle. Behavioral testing occurred 30 minutes after the injection. Drug test days were followed by a drug free day of no testing. Animals were then tested on the baseline schedule until performance stabilized before the next treatment. All drugs were purchased from Tocris

bioRxiv preprint doi: https://doi.org/10.1101/2024.05.09.593389; this version posted May 25, 2024. The copyright holder for this preprint (which was not certified by peer review) is the author/funder. This article is a US Government work. It is not subject to copyright under 17 USC 105 and is also made available for use under a CC0 license.

(Tocris Cookson Inc., Ellisville, Missouri, USA). Dimethyl sulfoxide (DMSO) and 2-hydroxypropyl-β-cyclodextrin (HBC) as dissolving agents were purchased from Sigma-Aldrich (St. Louis, MO, USA).

Following stable baseline performance, we examined the animals' choice of risk and safe options following changes to dopaminergic or adrenergic receptor activity **(Fig. 2A)**. Rats (n = 12) received injections of an adrenergic $\alpha_{2A}$-receptor agonist (guanfacine) and antagonist (yohimbine). Two weeks later, we induced stress in all animals by injecting them with a pharmacological stressor, FG 7142. We then sought to block or reverse the effects of the stressor by co-injecting it with the noradrenergic receptor specific drug. Since stress is also associated with dopamine (DA) release in various brain regions, for comparison, we repeated the procedure in another cohort of rats (n = 10) who received injections of a DA $D_1$ receptor agonist (SCH 23390) and antagonist (SKF 81297).

**Table 1. Summary details of drugs used in study**

| Drug description | Drug name | Doses (mg/kg) | Dissolving vehicle |
|---|---|---|---|
| $\alpha_{2A}$ adrenergic antagonist | Yohimbine | 1, 3, 6 | 50% sterile water in 0.9% saline |
| $\alpha_{2A}$ adrenergic agonist | Guanfacine | 0.1, 0.2, 0.5 | 0.9% saline |
| dopamine $D_1$ antagonist | SCH 23390 | 0.003, 0.007 | 0.9% saline |
| dopamine $D_2$ agonist | SKF 81297 | 0.1, 0.2, 0.5 | 0.9% saline |
| $GABA_A$ inverse agonist (pharmacological stressor) | FG 7142 | 1, 4 | 5-10% DMSO in 0.9% saline with 10% HBC |

DMSO, dimethyl sulfoxide; HBC, 2-hydroxypropyl-β-cyclodextrin,

**Fiber photometry: Viral injection and fiber implants**

In a subset of pretrained animals (n = 9), we injected, unilaterally, a genetically encoded fluorescent NE sensor, $GRAB_{NE}$ (pAAV-hSyn-GRAB_NE1m; Addgene #123308, gift from Yulong Li) into the basolateral amygdala (BLA), and a DA sensor, dLight (pAAV-syn-dLight1.3b; Addgene #135762, gift from Lin Tian) into the nucleus accumbens (NAc), to monitor NE and DA neural activity during stress induced decision-making. For all procedures involving local injections of injection and probe implantation, rats were anesthetized with isoflurane gas (5% induction, 2% maintenance) and secured in stereotaxic headholder (David Kopf Instruments, Tujanga, CA, USA). The scalp was retracted to expose the skull and craniotomies were made directly above the BLA (A/P -2.7 mm, M/L 4.9 mm, D/V -7.6) and NAc (A/P 1.7 mm, M/L 1.6 mm, D/V -7.3 mm). Viral injections were made using a pulled glass micropipette (WPI, USA) controlled by a Nanoliter 2020 injector (volume 300 nl at a rate of 100 nl/min). The virus was allowed to diffuse for 10 min before a slow withdrawal. Fiber optic cannulas (NA 0.66, 400-µm core diameter) were implanted

bioRxiv preprint doi: https://doi.org/10.1101/2024.05.09.593389; this version posted May 25, 2024. The copyright holder for this preprint (which was not certified by peer review) is the author/funder. This article is a US Government work. It is not subject to copyright under 17 USC 105 and is also made available for use under a CC0 license.

0.1 mm dorsal to the viral injection site (Doric Lenses, Canada). Cannulas were affixed with dental cement and stainless sterile screws to secure them in place.

**Fiber photometry recordings**

Following a minimum of two-weeks after surgery, rats were re-trained to acquire stable decision-making performance (~ 4 weeks). We first monitored NE and DA activity in the BLA and NAc, respectively while animals engaged in the decision-making task. Subsequently, we examined NE and DA responses following systemic injections of vehicle, yohimbine (1mg/kg) and FG7142 (4mg/kg).

Fiber photometry data were acquired with the RZ10X processor integrated with software Synapse v.96 (Tucker-Davis Technologies, Inc. USA). Light emitted from LED drivers integrated in the system (465 nm modulated at 330 Hz to excite dLight and GRAB-NE, and 405 nm modulated at 210 Hz for the isosbestic control) were transmitted through a Mini-cube fiber photometry apparatus (Doric Lenses) and low-autofluorescence patch-cord (400 µm core, 0.57 NA) connected to the implanted fiber-optic cannulas via a pigtailed rotary joint (Doric Lenses). The emitted signals were sent back to the Mini cube for filtration and detection by the integrated photosensors and demodulated in the Synapse software. In parallel, the RZ10X processor received time stamps of the behavioral events through a TTL breakout adapter (Lafayette Instruments, IN, USA). Raw fluorescence signals with behavioral time stamps were extracted into the Fiber photometry Modular Analysis Tool (pMAT) for further analysis [39]. Raw dLight or GRAB_NE signals were normalized to the isosbestic signal and transformed into delta F/F values. A custom-made R code and pMAT were used to calculate the delta F/F, Z-score and area under the curve (AUC) values used for analyses.

**Verification of fiber placement and viral expression**

Rats were perfused transcardially, with a working solution of phosphate buffer saline (1X PBS) followed by 4% paraformaldehyde (PFA) dissolved in PBS. The brains were extracted and post-fixed in 4% PFA overnight at 4 °C, and then dehydrated in 30% sucrose in PBS for a week. The brains were then cryo-sectioned to 40 µm thickness using a freezing microtome (Leica Biosystems, USA). Sections were mounted on glass slides with Vectashield antifade mounting medium (Vector Laboratories, USA). High resolution images were taken with a microscope scanner (Axio Scan 7; Zeiss). Animals with misplaced cannulas or viral expression were excluded from analysis.

**Statistical analysis**

The behavioral data were processed using custom-written programs in R and analyzed using SPSS Statistics 25.0 (IBM, Chicago, IL, USA). Incomplete data with sessions comprising less than 30% of free choices trials were not used for statistical analysis. All data were tested for normality and transformed accordingly before statistical significance testing. For comparisons between two groups *t*-tests were used. In cases when the data did not fit the assumptions of the test, the non-

bioRxiv preprint doi: https://doi.org/10.1101/2024.05.09.593389; this version posted May 25, 2024. The copyright holder for this preprint (which was not certified by peer review) is the author/funder. This article is a US Government work. It is not subject to copyright under 17 USC 105 and is also made available for use under a CC0 license.

parametric Mann–Whitney or Wilcoxon matched-pairs tests were used. For repeated measures ANOVA, data was assessed for homogeneity of variance using Mauchly's sphericity test. When this requirement was violated for a repeated measures design, the F term was tested against degrees of freedom corrected by Greenhouse–Geisser to provide a more conservative p value for each F ratio. Otherwise, nonparametric Friedman's test ($\chi^2$) was applied with differences compared with posthoc Wilcoxon signed-rank tests (Z) adjusted with a Bonferroni correction. Pearson correlation (r) was used to describe the linear relationship between two correlated variables.

bioRxiv preprint doi: https://doi.org/10.1101/2024.05.09.593389; this version posted May 25, 2024. The copyright holder for this preprint (which was not certified by peer review) is the author/funder. This article is a US Government work. It is not subject to copyright under 17 USC 105 and is also made available for use under a CC0 license.

# Figure S2

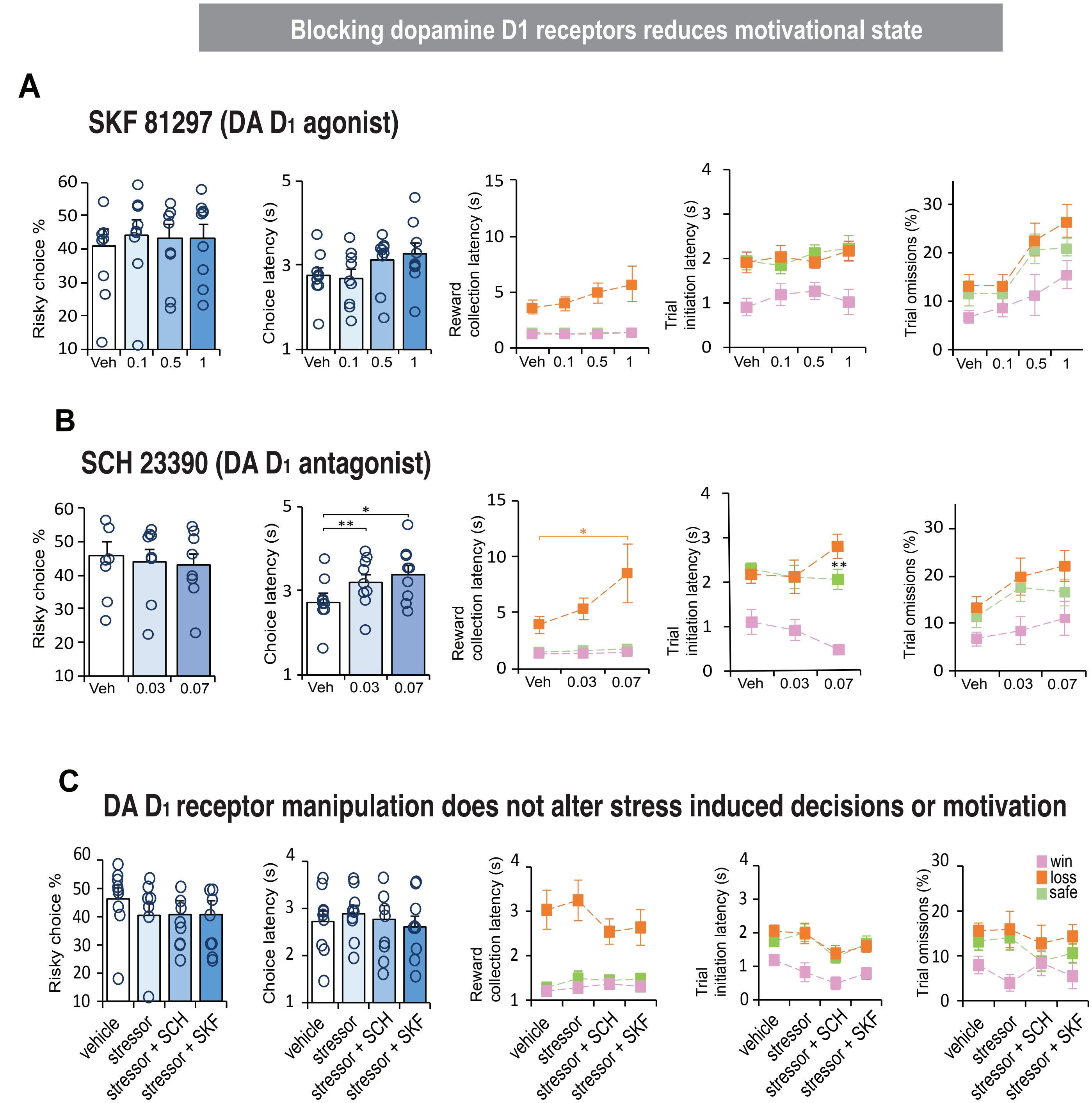

# Figure S3

## Photometry fiber placements

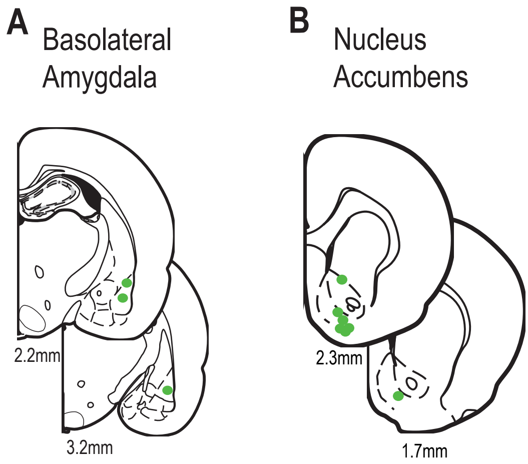

bioRxiv preprint doi: https://doi.org/10.1101/2024.05.09.593389; this version posted May 25, 2024. The copyright holder for this preprint (which was not certified by peer review) is the author/funder. This article is a US Government work. It is not subject to copyright under 17 USC 105 and is also made available for use under a CC0 license.

# Figure S4

## Dynamics of NE release in the BLA during reward collection

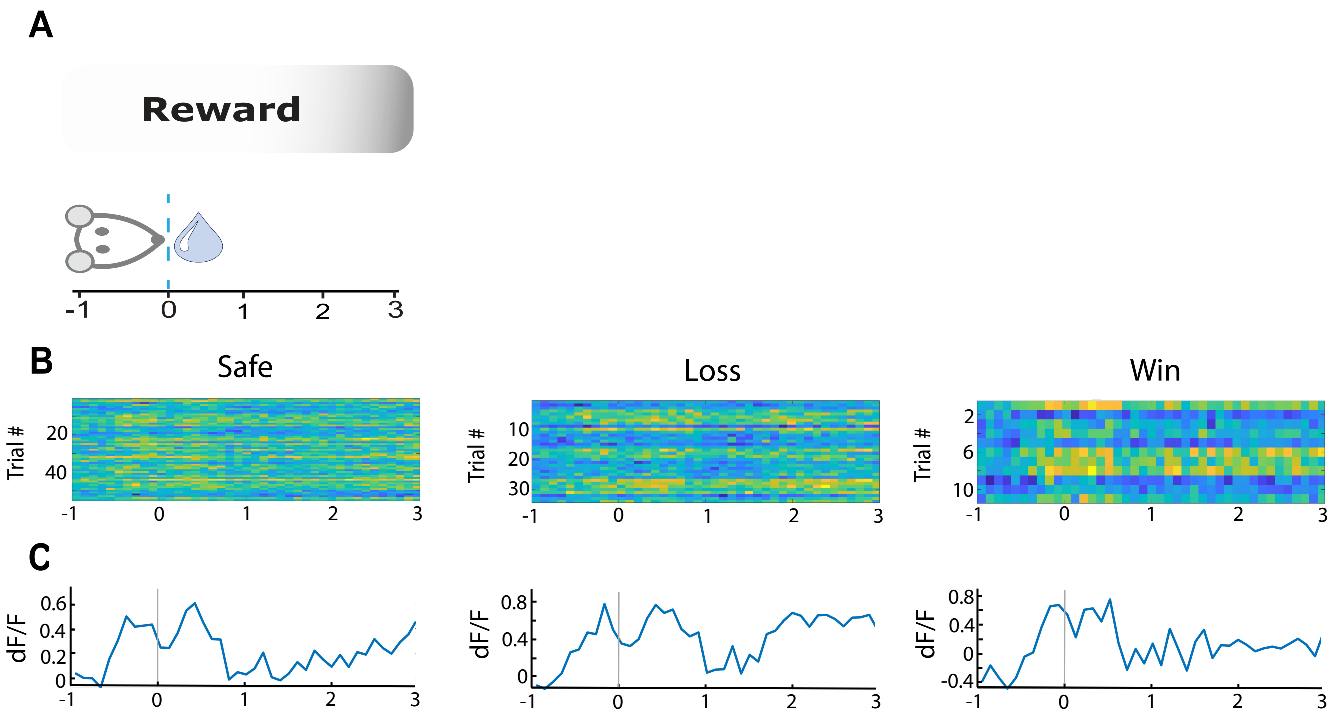


## Dynamics of DA release in the NAc when making a choice

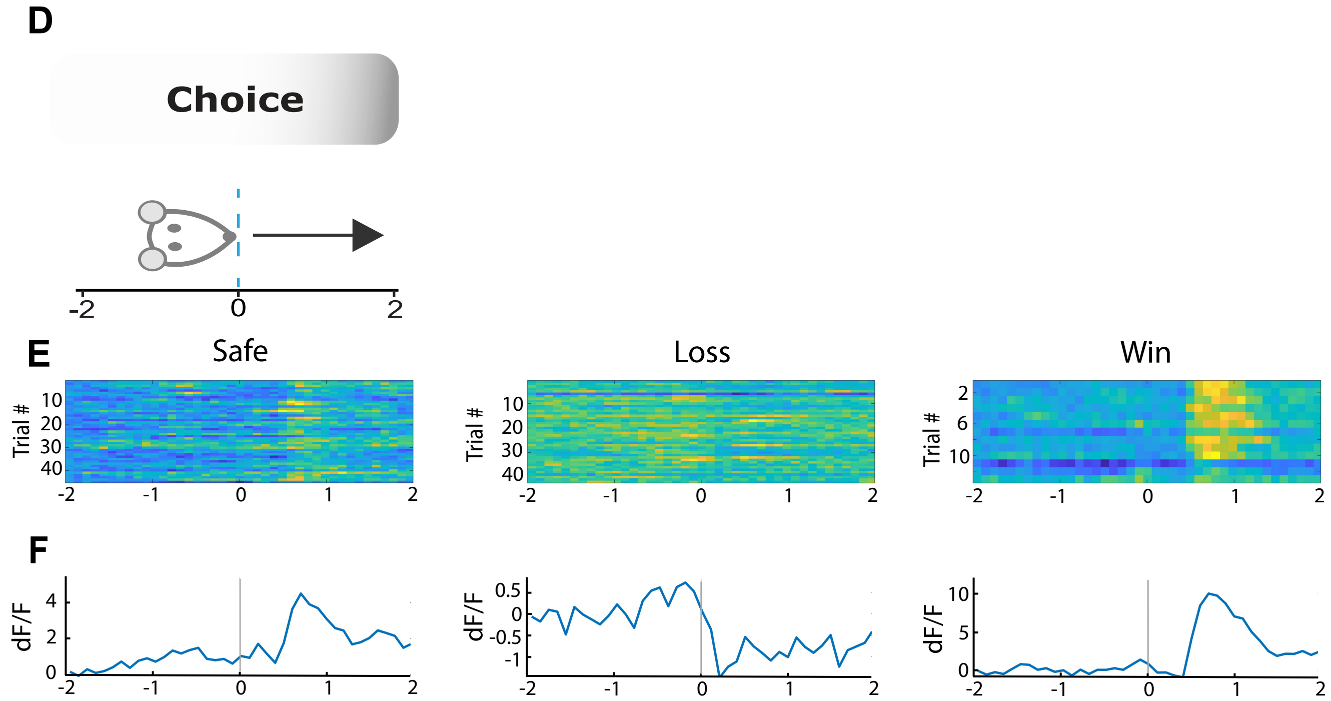

# Figure S5

bioRxiv preprint doi: https://doi.org/10.1101/2024.05.09.593389; this version posted May 25, 2024. The copyright holder for this preprint (which was not certified by peer review) is the author/funder. This article is a US Government work. It is not subject to copyright under 17 USC 105 and is also made available for use under a CC0 license.

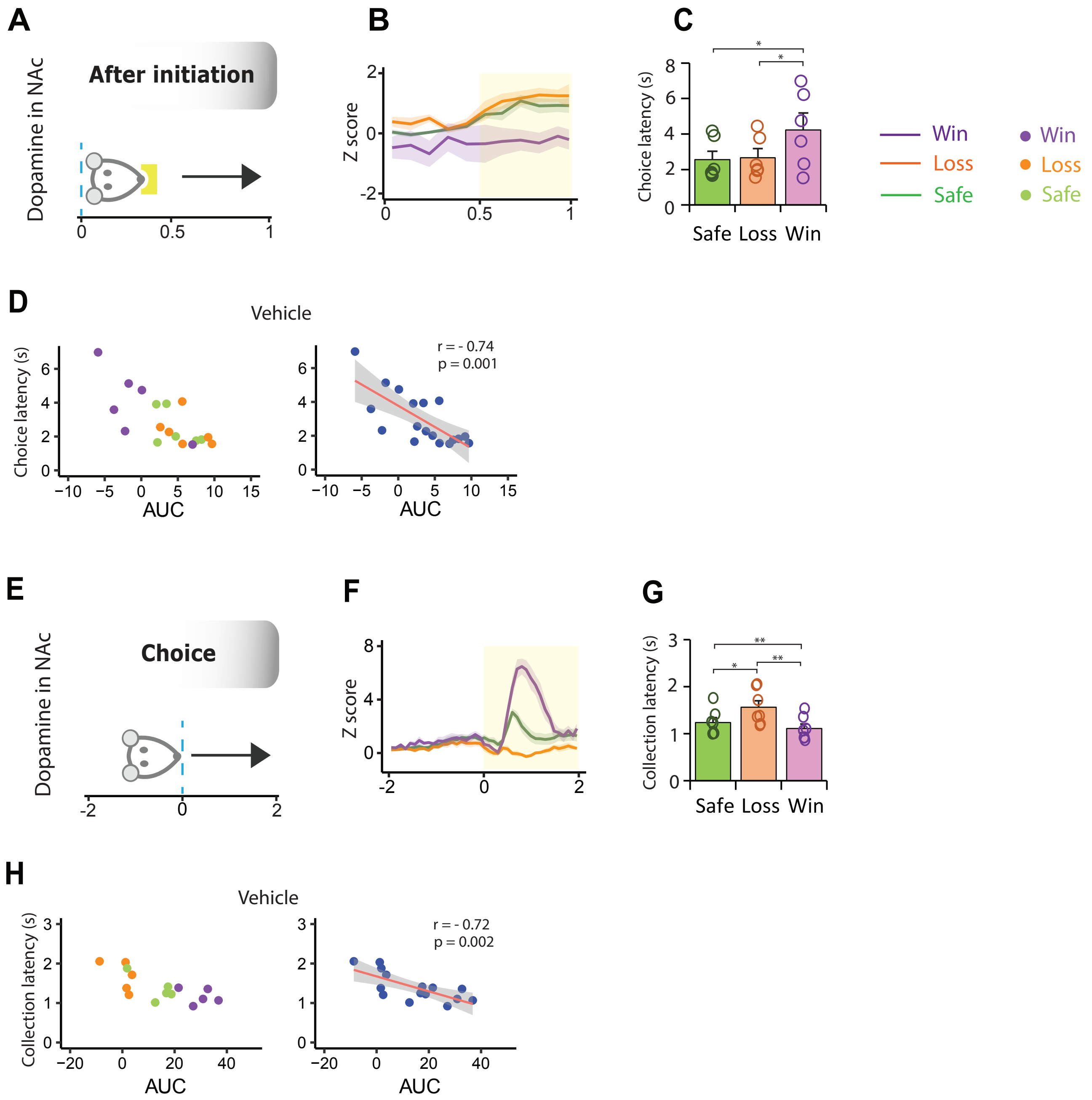

Supplement: Supplementary file 1 [file NIHPP2024.05.09.593389v2-supplement-1.pdf]
