# Supplementary material for: Noradrenergic modulation of stress induced catecholamine release: Opposing influence of FG7142 and yohimbine
Source: bioRxiv. 2024 May 9:2024.05.09.593389. Preprint. [Version 1] doi: 10.1101/2024.05.09.593389 (PMC11100835; doi:10.1101/2024.05.09.593389)

Figure S1

Photometry fiber placements

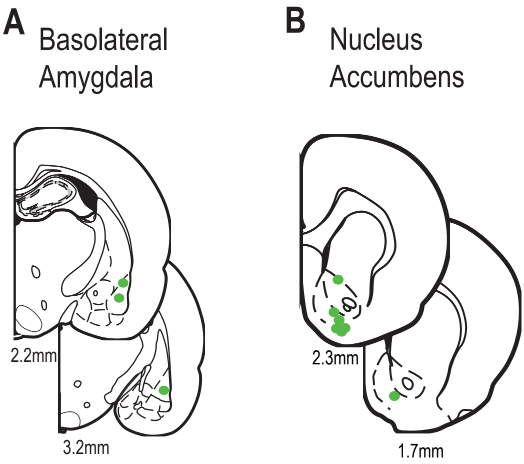

bioRxiv preprint doi: https://doi.org/10.1101/2024.05.09.593389; this version posted May 9, 2024. The copyright holder for this preprint (which was not certified by peer review) is the author/funder. This article is a US Government work. It is not subject to copyright under 17 USC 105 and is also made available for use under a CC0 license.

Figure S2

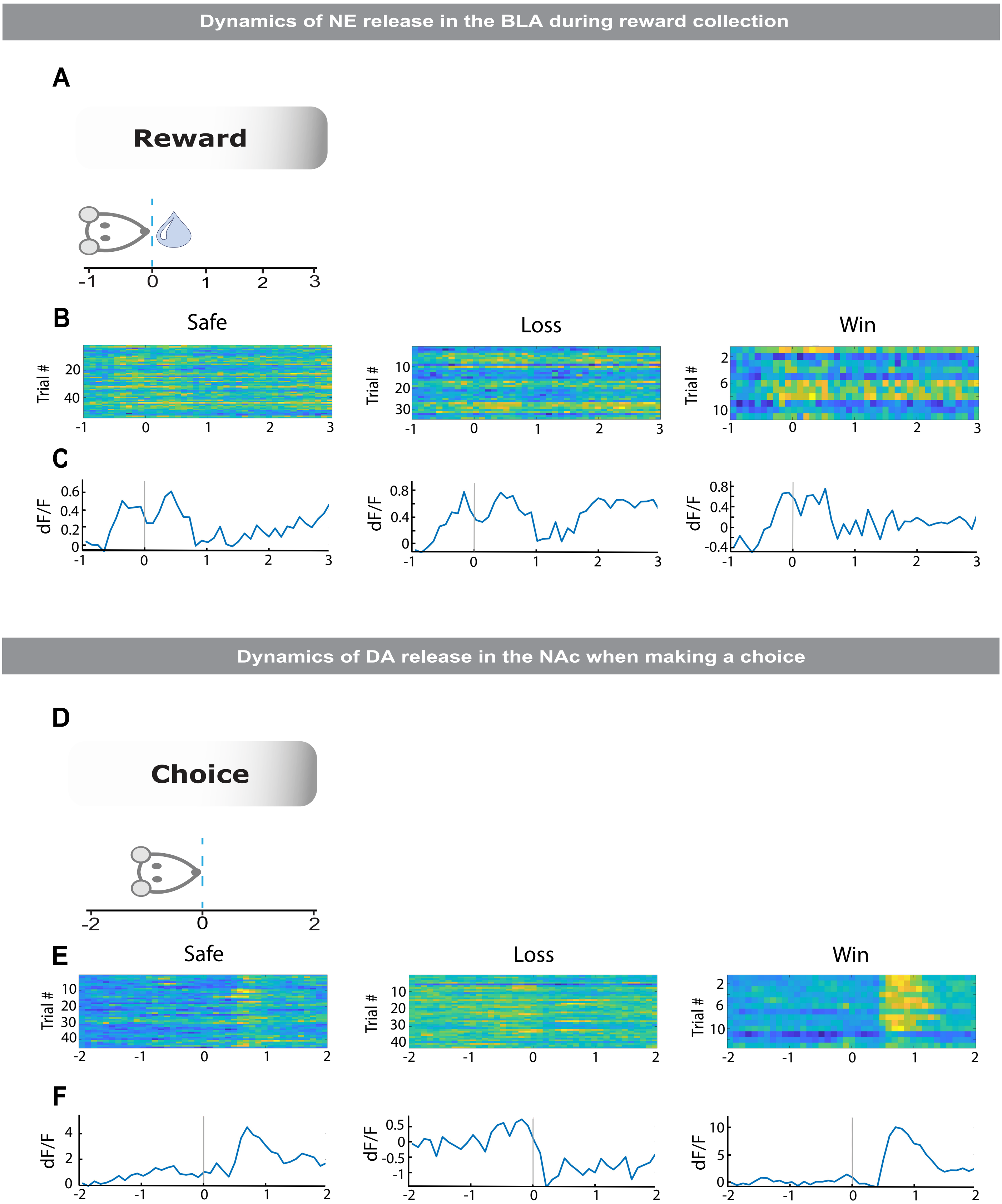

bioRxiv preprint doi: https://doi.org/10.1101/2024.05.09.593389; this version posted May 9, 2024. The copyright holder for this preprint (which was not certified by peer review) is the author/funder. This article is a US Government work. It is not subject to copyright under 17 USC 105 and is also made available for use under a CC0 license.

Figure S3

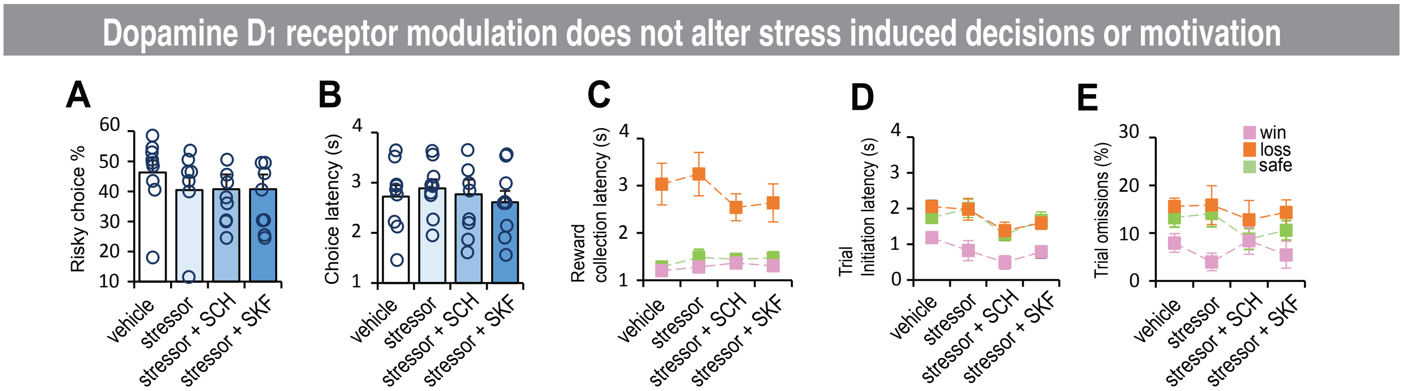

Figure S4

bioRxiv preprint doi: https://doi.org/10.1101/2024.05.09.593389; this version posted May 9, 2024. The copyright holder for this preprint (which was not certified by peer review) is the author/funder. This article is a US Government work. It is not subject to copyright under 17 USC 105 and is also made available for use under a CC0 license.

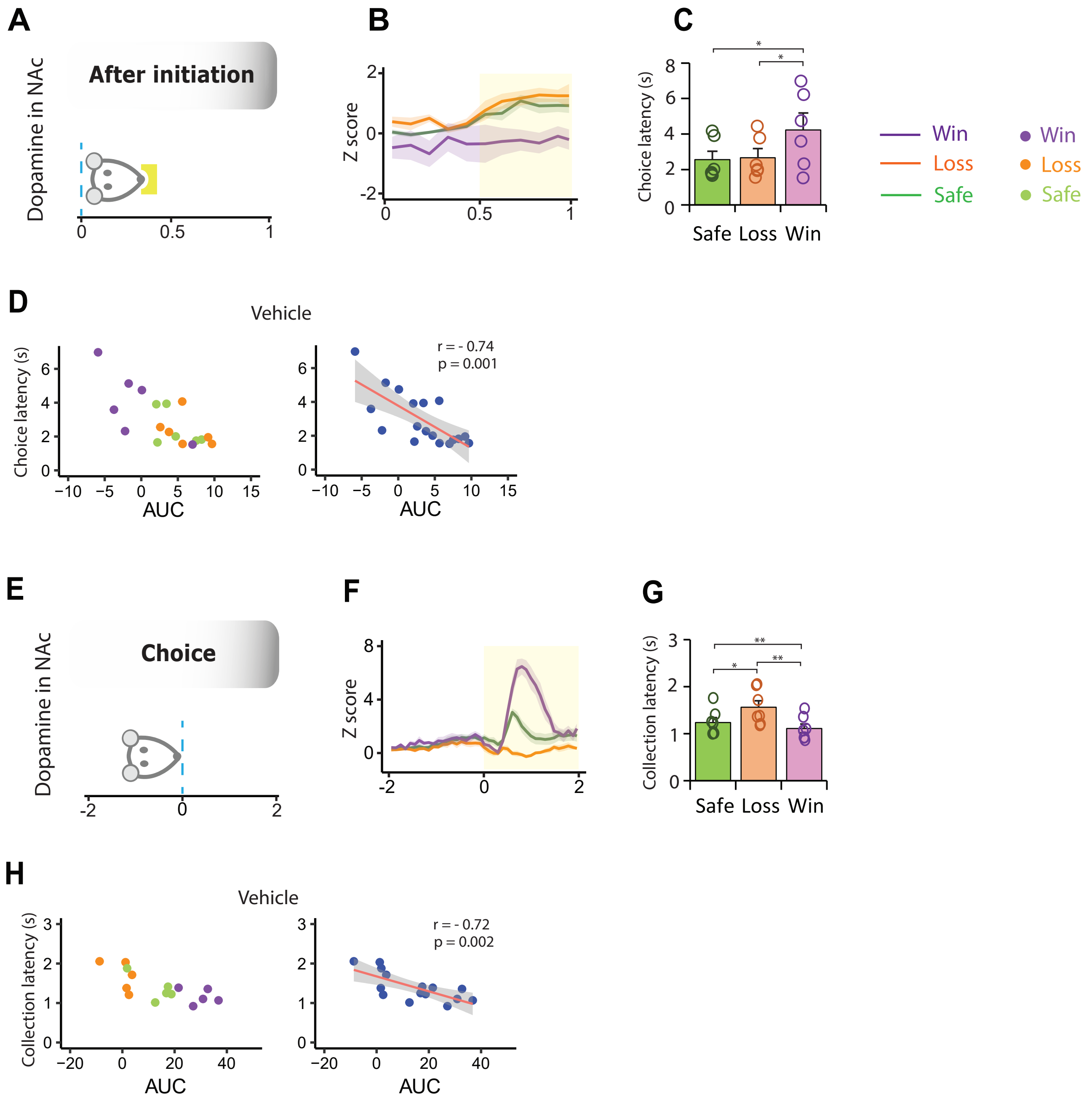

Supplement: Supplementary file 1 [file NIHPP2024.05.09.593389v1-supplement-1.pdf]
